# Is Our Natural Food Our Homeostasis? Array of a Thousand Effect-Directed Profiles of 68 Herbs and Spices

**DOI:** 10.3389/fphar.2021.755941

**Published:** 2021-12-09

**Authors:** Tamara Schreiner, Dorena Sauter, Maren Friz, Julia Heil, Gertrud Elisabeth Morlock

**Affiliations:** Institute of Nutritional Science, Chair of Food Science, and TransMIT Center for Effect-Directed Analysis, Justus Liebig University Giessen, Giessen, Germany

**Keywords:** botanical, effect-directed analysis, 8D hyphenation, high-performance thin-layer chromatography, high-performance liquid chromatography, mass spectrometry

## Abstract

The beneficial effects of plant-rich diets and traditional medicines are increasingly recognized in the treatment of civilization diseases due to the abundance and diversity of bioactive substances therein. However, the important active portion of natural food or plant-based medicine is presently not under control. Hence, a paradigm shift from quality control based on marker compounds to effect-directed profiling is postulated. We investigated 68 powdered plant extracts (botanicals) which are added to food products in food industry. Among them are many plants that are used as traditional medicines, herbs and spices. A generic strategy was developed to evaluate the bioactivity profile of each botanical as completely as possible and to straightforwardly assign the most potent bioactive compounds. It is an 8-dimensional hyphenation of normal-phase high-performance thin-layer chromatography with multi-imaging by ultraviolet, visible and fluorescence light detection as well as effect-directed assay and heart-cut of the bioactive zone to orthogonal reversed-phase high-performance liquid chromato-graphy−photodiode array detection−heated electrospray ionization mass spectrometry. In the non-target, effect-directed screening via 16 different on-surface assays, we tentatively assigned more than 60 important bioactive compounds in the studied botanicals. These were antibacterials, estrogens, antiestrogens, androgens, and antiandrogens, as well as acetylcholinesterase, butyrylcholinesterase, α-amylase, α-glucosidase, β-glucosidase, β-glucuronidase, and tyrosinase inhibitors, which were on-surface heart-cut eluted from the bioautogram or enzyme inhibition autogram to the next dimension for further targeted characterization. This biological-physicochemical hyphenation is able to detect and control active mechanisms of traditional medicines or botanicals as well as the essentials of plant-based food. The array of 1,292 profiles (68 samples × 19 detections) showed the versatile bioactivity potential of natural food. It reveals how efficiently and powerful our natural food contributes to our homeostasis.

## 1 Introduction

Herbs and spices are widely used for nutrition, flavoring, cosmetics, dyeing, or fragrances (Guldiken et al., 2018). They are also applied in medicine due to their known beneficial effects on human health (Yuan et al., 2016; Caesar et al., 2019), inspired by traditional healers who have used botanical extracts since ancient times (Belwal et al., 2018b). The knowledge of biologically active plants, their harvesting, production, preparation, and administration has been passed down through thousands of years of traditional medicine (Yuan et al., 2016). Particularly phenols were reported to have antibacterial, antiviral, and antioxidant effects, as well as the ability to modulate enzyme activity and transduction pathways (Krüger et al., 2017; Tresserra-Rimbau et al., 2018). Some studies have quantified the total amount of healthful constituents in herbal extracts and calculated the recommended intake of antioxidants from culinary herbs (Halvorsen et al., 2002; Wojdylo et al., 2007). However, their multifactorial relevance in homeostasis is underexplored. It is evident that the use of the whole natural plant extract is more powerful for homeostasis due to the versatility of the gentle mechanisms of active compounds than the use of isolated compounds (Morlock and Heil, 2020).

In a typical screening for potential drug candidates, plant extracts are currently freed from assay-interfering tannins by solid-phase extraction, separated with an HPLC gradient (42 min/sample including equilibration), and collected in fractions, which are screened for bioactivity in a microtiter plate assay (Kongstad et al., 2015). Therefore, bioactivity can only be assigned to a fraction containing several analytes via a costly and time-consuming workflow, which subsequently requires analytical separation and testing of each peak to assign the individual bioactive compounds (Caesar et al., 2019). In routine, there has only been a little progress in non-target screening of food for bioactive compounds at an affordable price. Most methods deal with illicit additions, organic contaminants (Fu et al., 2017), adulterated foods (Díaz et al., 2012), and migrants from packaging (Rusko et al., 2020; Su et al., 2020). Also, generic chromatography-based high-resolution mass spectrometric methods were examined to cover as many substances as possible within a single analysis (Díaz et al., 2012). However, one drawback is the high load of interfering matrix caused by the diversity and abundance of substances in such natural products as spices and herbs (Caesar et al., 2019; Morlock and Heil, 2020). Elaborate sample preparation (which is selective and error-prone) would otherwise limit the validity and significance of the results. The state of the art is setting an intensity threshold and focusing on highly abundant signals (Wu et al., 2016). But even the smallest signal can have an important biological effect. Ignoring minor signals from the set instrumental threshold will produce grossly negligent results. Moreover, compounds may not ionize well or at all with standard settings of mass spectrometric recording. That is why routine analysis of natural extracts is still tailored and limited to marker compounds. However, the important active portion of natural food needs to be under (analytical) control, which is presently not the case.

To overcome these limitations and expand the analytical toolbox, a high-throughput eight-dimensional (8D) hyphenat-ion was recently developed, and its proof of principle was shown for cinnamon samples detected with an antibacterial bioassay (Schreiner and Morlock, 2021). It demonstrated the information gained by combining effect-directed assays (EDA) with normal-phase high-performance thin-layer chromatography including multi-imaging by ultraviolet, visible, and fluorescence light detection (NP-HPTLC−UV/Vis/FLD) (Morlock, 2021). Heart-cut elution and transfer of the bioactive compound zone to an orthogonal reversed-phase high-performance liquid chromatography (RP-HPLC) system was exploited to separate potentially coeluting bioactive substances. The subsequent photodiode array detection (DAD) and heated electrospray ionization mass spectrometry (HESI-MS) were used for additional straightforward characterization of the bioactive substances. The advantage of NP-HPTLC−UV/Vis/FLD−EDA−heart-cut RP-HPLC−DAD−HESI-MS is that it prioritizes and reduces the thousands of compounds in such natural samples to the most important bioactive compounds. As the previously developed hyphenation was only shown for cinnamon and one antibacterial bioassay, this study intended to examine the influence of 68 different plant matrices and 16 different assays on the robustness of the new 8D hyphenation. It was of interest to prove its universal validity and significance, to figure out potential limitations, and to verify its suitability as generic activity screening. Such straightforward effect-directed profiling could be applied to reveal, understand, and control the mode of action of traditional medicines, botanicals, and plant-based food.

## 2 Materials and Methods

### 2.1 Chemicals and Materials

Purity grades were listed when available. All salts were of p. a. quality and water free unless stated otherwise. Ethanol, toluene (all solvents of chromatography grade), bovine serum albumin (BSA, fraction V, ≥98%), dipotassium hydrogen phosphate (K_2_HPO_4_, ≥99%), sodium dihydrogen phosphate monohydrate (NaH_2_PO_4_ ∙ H_2_O, ≥98%), glycerol (Rotipuran, 86%), potassium dihydrogen phosphate (KH_2_PO_4_, ≥99%), dipotassium hydrogen phosphate trihydrate (K_2_HPO_4_ ∙ 3 H_2_O, ≥99%), sodium hydroxide (NaOH, ≥98%), disodium hydrogen phosphate (Na_2_HPO_4_, ≥99%), potassium chloride (KCl, 98.5%), polyethylene glycol (PEG) 8000 (Ph. Eur.), kojic acid (>98%), acetic acid (100%), sulfuric acid (96%), hydrochloric acid (37%, HCl, purest), citric acid (p. a.), 3-[4,5-dimethylthiazol-2-yl]-2,5-diphenyltetrazolium bromide (MTT, ≥98%), 3-[(3cholamidopropyl) dimethylammonio]-1-propanesulfonate (CHAPS, ≥98%), dimethyl sulfoxide (DMSO), and tris(hydroxymethyl)aminomethane (TRIS, ≥99.9%) were obtained from Carl Roth, Karlsruhe, Germany. Diammonium hydrogen phosphate ([NH_4_]_2_HPO_4_, ≥99%) was purchased from Acros Organics, Morris Plains, NJ, United States. Butyrylcholinesterase (BChE) from equine serum (≥140 U/mg) was provided by SERVA, Heidelberg, Germany. Acarbose (≥95%), α-glucosidase from *Saccharomyces cerevisiae* (1,000 U/vial), tyrosinase from mushroom (≥1,000 U/mg, 25 kU/vial), β-glucuronidase from *Escherichia coli* (5,000 U/vial), acetylcholinesterase (AChE) from *Electrophorus electricus* (≥245 U/mg, 10 kU/vial), peptone from casein (for microbiology), sodium acetate, sodium chloride (NaCl), Müller-Hinton broth (for microbiology), d-(+)-glucose (99.5%), rivastigmine (≥98%), imidazole (≥99.5%), copper sulfate, 7-hydroxy-4-methylcoumarin (4-methylumbelliferone, >98%), yeast nitrogen base without amino acids (for molecular biology), quercitin-3-*O*-glucoside (≥90%), liquiritigenin (≥97%), naringenin (≥95%), syringic acid (≥95%), pinobanksin (≥95%), sodium hydrogen carbonate (99.7%), lysogeny broth (containing 5 mg/ml sodium chloride) powder, ampicillin sodium salt, α-amylase from hog pancreas (50 U/mg), Gram’s iodine solution (for microscopy) and testosterone (≥99%) were delivered by Sigma-Aldrich, Steinheim, Germany. 2-Naphthyl-β-d-glucopyranoside (95%) and β-glucosidase from almonds (3,040 U/mg) were provided by ABCR, Karlsruhe, Germany. 1-Naphthyl acetate (≥98%) and 2-naphthyl-α-d-glucopyranoside were obtained from AppliChem, Darmstadt, Germany. Fast Blue B salt (95%) was purchased from MP Biomedicals, Eschwege, Germany. 5-Bromo-4-chloro-3-indolyl-β-d-glucopyranosid-uronic sodium salt was obtained from Carbosynth, Compton-Berkshire, United Kingdom. Methanol (MS quality) and formic acid (99%) were delivered from VWR, Darmstadt, Germany. d-Saccharolactone and (2S)-2-amino-3-(3,4-dihydroxyphenyl) propionic acid (levodopa) was obtained from Santa Cruz Biotechnology, Dallas, TX, United States. 17-β-Estradiol (98.5%) was obtained from Dr. Ehrenstorfer, Augsburg, Germany. Ethyl acetate (≥99.8%) and yeast extract powder (for microbiology) were purchased from Th. Geyer, Renningen, Germany. The medium for the Gram-negative, naturally luminescent marine *Aliivibrio fischeri* bacteria (DSM-7151, German Collection of Microorganisms and Cell Cultures, Berlin, Germany) is listed elsewhere (European Committee for Standardization, 2009). Gram-positive soil bacteria *Bacillus subtilis* subsp. *spizizenii* (DSM-618), magnesium sulfate heptahydrate (MgSO_4_ ∙ 7 H_2_O, 99.5%), citric acid monohydrate (≥99.5%), 4-methyl-umbelliferyl-β-d-galactopyranoside, phosphate-buffered saline (without Ca^2+^), soluble starch, as well as HPTLC plates silica gel 60 F_254_ MS-grade and HPTLC plates silica gel 60 (both 20cm × 10 cm) were provided by Merck, Darmstadt, Germany. Bidistilled water was prepared by a Heraeus Destamat Bi-18 E (Thermo Fisher Scientific, Dreieich, Germany). *Saccharomyces cerevisiae* BJ 1991, equipped with the human androgen receptor, S9 enzyme mixture (from rat liver), nicotinamide adenine dinucleotide phosphate (NADP), and glucose 6-phosphate were delivered by Xenometrix, Allschwil, Switzerland. Additional chemicals and reagents used for planar yeast ant-/agonistic androgen/estrogen screens were reported elsewhere (Klingelhöfer and Morlock, 2015; Klingelhöfer et al., 2020). The *Saccharomyces cerevisiae* cells equipped with the hERβ were obtained from the Erwin Herberle-Bors, University of Vienna, Austria (Kirchmayer, 2009). Reference substances acacetin (99%), eriocitrin (96%), naringin (92%), ginkgolide A (99%) and B (99%), isorhamnetin (99%), liquiritin apioside (≥95%), hesperidin (>96%), (−)-epicatechin (100%), (+)-catechin (98%), rutin (90%), and meranzin (98%) were obtained from PhytoLab, Vestenbergsgreuth, Germany. Rosmaric acid (≥98%), galangin (≥98%), chlorogenic acid, kaempferol, and daidzein were delivered by Cayman Chemical, Ann Arbor, MI, United States. Glycyrrhizic acid and 4-nitroquinoline-1-oxide (98%) were purchased from TCI, Eschborn, Germany. The strain TA1535 of *Salmonella typhimiurium* (genetically modified to contain the plasmid pSK1002) was purchased as cryostock from Trinova Biochem, Giessen, Germany. Resorufin-β-d-galactopyranoside was obtained from Toronto Research Chemicals, Toronto, Canada.

### 2.2 Standard Solutions and Sample Preparation

Standards solutions were prepared in methanol (1 mg/ml). Samples were obtained as dried, homogenized (mostly aqueous) extracts from Martin Bauer Group, Vestenbergsgreuth, Germany. For a 10% extract solution, an aliquot (0.5 g, Table 1) of each botanical powder was suspended in 5 ml methanol, ultra-sonicated for 30 min (Sonorex Digiplus, Bandelin, Berlin, Germany), and centrifuged at 3,000 × *g* for 15 min (Labofuge 400, Heraeus, Hanau, Germany). Each supernatant was transferred in an autosampler vial. Some extracts were additionally filtered (Table 1, marked*) through a 0.45 µm polytetrafluoroethylene filter (VWR, Darmstadt, Germany).

**TABLE 1:** Compilation of 68 botanicals, including botanical name, plant part, and sample weights (W) extracted with 5 ml methanol (*filtered through 0.45 µm PTFE filter).

No	Common name	Botanical name	Plant part	W [mg]
**1**	Acerola	*Malpighia glabra* L [Malphighiaceae]	fruits	501.5
**2**	Horehound, white	*Marrubium vulgare* L. [Lamiaceae]	herb	500.1
**3**	Apple*	*Malus sylvestris* (L.) Mill. [Rosaceae]	peel	500.7
**4**	Artichoke, globe	*Cynara cardunculus* subsp. *scolymus* (L.) [Asteraceae]	leaves	501.3
**5**	Basil	*Ocimum basilicum* L. [Lamiaceae]	herb	500.6
**6**	Fenugreek	*Trigonella foenum-graecum* L. [Fabaceae]	seeds	499.9
**7**	Stinging nettle*	*Urtica dioica* L. [Urticaceae]	leaves	501.5
**8**	Blackberry	*Rubus fruticosus* L. [Rosaceae]	leaves	500.6
**9**	*Eucalyptus*	*Eucalyptus globulus* Labill. [Myrtaceae]	leaves	499.7
**10**	Fennel	*Foeniculum vulgare* Mill. [Apiaceae]	fruits	499.9
**11**	Fruit tea, yellow	not available	unknown	501.3
**12**	Fruit tea, red	not available	unknown	502.6
**13**	Galangal	*Alpinia officinarum* Hance. [Zingiberaceae]	roots	501.8
**14**	Ginkgo	*Ginkgo biloba* L. [Ginkgoaceae]	leaves	502.7
**15**	Ginseng	*Panax ginseng* C.A.Mey. [Araliaceae]	roots	502.3
**16**	Guarana	*Paullinia cupana* Kunth [Sapindaceae]	seeds	498.8
**17**	Dog rose	*Rosa canina* L. [Rosaceae]	fruits	501.0
**18**	Blueberry, European	*Vaccinium myrtillus* L. [Eriaceae]	fruits	501.2
**19**	*Hibiscus*	*Hibiscus rosa-sinensis* L. [Malvaceae]	blossoms	499.6
**20**	Raspberry	*Rubus idaeus* L. [Rosaceae]	juice concentrate from fruits	503.0
**21**	Elderberry	*Sambucus nigra* L. [Adoxaceae]	fruits	501.4
**22**	Elder flower	*Sambucus nigra* L. [Adoxaceae]	blossoms	502.5
**23**	Honeybush*	*Cyclopia genistoides* (L.) R.Br. [Fabaceae]	leaves, branches, blossoms	499.3
**24**	Hop	*Humulus lupulus* L. [Cannabaceae]	blossoms	502.1
**25**	Ginger	*Zingiber officinale* Roscoe [Zingiberaceae]	roots	499.0
**26**	Jasmine*	*Jasminum officinale* L. [Oleaceae]	blossoms	499.2
**27**	Cassis	*Ribes nigrum* L. [Grossulariaceae]	juice concentrate from fruits	500.7
**28**	Chamomile	*Matricaria chamomilla* L. [Asteraceae]	blossoms	499.3
**29**	Cardamom*	*Elettaria cardamomum* (L.) Maton [Zingiberaceae]	fruits	499.6
**30**	Garlic	*Allium sativum* L. [Amaryllidaceae]	bulbs	499.9
**31**	Kola*	*Cola nitida* (Vent.) Schott and Endl. [Malvaceae]	seeds	500.8
**32**	Coriander	*Coriandrum sativum* L. [Apiaceae]	fruits	501.3
**33**	Caraway	*Carum carvi* L. [Apiaceae]	fruits	500.0
**34**	Lovage	*Levisticum officinale* W.D.J.Koch [Apiaceae]	roots	499.6
**35**	Marjoram	*Origanum majorana* L. [Lamiaceae]	herb	502.4
**36**	Yerba mate*	*Ilex paraguariensis* A.St.-Hil. [Aquifoliaceae]	leaves, roasted	499.6
**37**	Yerba mate	*Ilex paraguariensis* A.St.-Hil. [Aquifoliaceae]	leaves	500.2
**38**	Lemon balm	*Melissa officinalis* L. [Lamiaceae]	leaves	500.6
**39**	Clove^*^	*Syzygium aromaticum* (L.) Merr. and L.M.Perry [Myrtaceae]	flower buds	501.9
**40**	Orange	*Citrus × aurantium* L. [Rutaceae]	blossoms	499.7
**41**	Orange	*Citrus × aurantium* L. [Rutaceae]	peel	501.1
**42**	Oregano	*Origanum vulgare* L. [Lamiaceae]	herb	501.5
**43**	Passionflower	*Passiflora incarnata* L. [Passifloraceae]	blossoms	501.1
**44**	Peppermint	*Mentha × piperita* L. [Lamiaceae]	leaves	500.3
**45**	Rooibos*	*Aspalathus linearis* (Burm.f.) R.Dahlgren [Fabaceae]	leaves	500.7
**46**	Rosemary*	*Salvia Rosmarinus* Spenn. [Lamiaceae]	leaves	500.9
**47**	Sage	*Salvia officinalis* L. [Lamiaceae]	leaves	499.9
**48**	Sea buckthorn	*Hippophae rhamnoides* L. [Elaeagnaceae]	fruits	501.9
**49**	Horsetail	*Equisetum arvense* L. [Equisetaceae]	herb	499.3
**50**	Yarrow*	*Achillea millefolium* L. [Asteraceae]	herb	501.6
**51**	Celeriac	*Apium graveolens* L. [Apiaceae]	bulb	501.3
**52**	Coneflower	*Echinacea angustifolia* DC. [Asteraceae]	herb and roots	499.1
**53**	Plantain	*Plantago lanceolata* L. [Plantaginaceae]	leaves	500.5
**54**	Star anise	*Illicium verum* Hook.f. [Schisandraceae]	fruits	500.3
**55**	Licorice	*Glycyrrhiza glabra* L. [Fabaceae]	roots	500.3
**56**	Siberian ginseng	*Eleutherococcus senticosus* (Rupr. and Maxim.) Maxim. [Araliaceae]	roots	503.4
**57**	Thyme	*Thymus vulgaris* L. [Lamiaceae]	herb	499.6
**58**	Grape*	*Vitis vinifera* L. [Vitaceae]	seed	499.9
**59**	Grape	*Vitis vinifera* L. [Vitaceae]	peel	499.7
**60**	Juniper	*Juniperus communis* L. [Cupressaceae]	fruits	501.5
**61**	Grape	*Vitis vinifera* L. [Vitaceae]	leaves	501.2
**62**	Hawthorn	*Crataegus* sp. [Rosaceae]	leaves and blossoms	499.7
**63**	Hawthorn leaves (Batch 1)	*Crataegus* sp. [Rosaceae]	leaves	501.8
**64**	Hawthorn leaves (Batch 2)	*Crataegus* sp. [Rosaceae]	leaves	499.9
**65**	Chicory	*Cichorium intybus* L. [Asteraceae]	roots	501.1
**66**	Cinnamon	*Cinnamomum verum* J.Presl [Lauraceae]	bark	501.5
**67**	Lemon peel	*Citrus × limon* (L.) Osbeck [Rutaceae]	peel	500.7
**68**	Lemon verbena	*Aloysia citridora* Paláu [Verbenaceae]	leaves	500.4

### 2.3 HPTLC–UV/Vis/FLD

Plates were pre-washed with methanol—water (4:1 *V/V*), dried in an oven (Memmert, Schwabach, Germany) for 20 min at 110°C (Morlock, 2014), and stored wrapped in aluminum foil. All botanical extracts (4 µL/band) were applied as 6 mm bands on a pre-washed plate (Automatic TLC Sampler 4, CAMAG, Muttenz, Switzerland). The plate was developed up to a migration distance of 70 mm with 7 ml ethyl acetate—toluene—formic acid—water (16:4:3:2 *V/V/V/V*) (Krüger et al., 2017). Separation was performed in a twin trough chamber (20cm × 10 cm, CAMAG) followed by drying for 4 min with a stream of cold air (hair dryer) and for 20 min in a laminar flow of air (Automated Development Chamber 2, CAMAG). The developed plates were documented at Vis, UV 254 nm, and FLD 366 nm (TLC Visualizer 2, CAMAG). The software winCATS (version 1.4.7.2018) or visionCATS (version 2.5.18262.1, both CAMAG) controlled the instruments.

### 2.4 HPTLC–EDA

For bioprofiling, 16 silica gel 60 F_254_ MS-grade chromatograms were prepared. The buffer and assay solutions were piezoelectrically sprayed (Derivatizer, CAMAG) if not stated otherwise. To remove acidic traces left on the planar chromatogram (which can interfere with pH-sensitive bioassays), the chromatogram was neutralized with 1.5 ml phosphate buffer (80 mg/ml Na_2_HPO_4_, pH 7.5 adjusted with NaOH; yellow/green nozzle, level 6) for enzymatic and bacterial assays, 1.4 ml citrate buffer (6 mg/ml citric acid monohydrate, 10 mg/ml Na_2_HPO_4_, adjusted to pH 12 with NaOH, yellow ultra-nozzle, level 2) (Klingelhöfer et al., 2020) for the hormonal-effective bioassays, 1.25 ml sodium bicarbonate buffer (2.5%, yellow nozzle, level 3) for α-amylase bioassay or twice with 2.8 ml sodium bicarbonate buffer for SOS-Umu-C bioassay (neutralization procedure for SOS-Umu-C bioassay was investigated during this study, Supplementary Figure S1). The moist chromatogram was dried as mentioned in 2.3. A positive control was applied at three different concentrations at the top plate edge to verify the proper bioassay performance. The assay solutions/suspensions were applied as follows. For incubation, the plates were horizontally placed in a moistened polypropylene KIS box (26.5 cm × 16 cm × 10 cm, ABM, Wolframs-Eschenbach, Germany) pre-saturated with 30 ml water at 37°C (30°C for hormonal-effective bioassays) for 30 min. The procedure was documented at FLD 366 nm and white light illumination in transmission, reflection, and reflection/transmission mode.

#### 2.4.1 *Bacillus subtilis* Bioassay

For the Gram-positive *B. subtilis* inhibition bioassay, 80 µl of stock solution was suspended in 20 ml Müller-Hinton Broth and incubated overnight at 37°C. Before usage, the cell number was determined using a spectrophotometer (M501, Camspec, Garforth, United Kingdom) at 600 nm. At an optical density (OD_600_) between 0.8 and 1.1, the culture was ready to use for EDA. An aliquot of the bacteria suspension (2 ml) was sprayed on the planar chromatogram (red nozzle, level 6) (Morlock et al., 2021b). The plate was incubated at 37°C for 2 h. As substrate solution (2 mg/ml), MTT was freshly prepared in phosphate-buffered saline. After the application of 250 µl substrate solution (blue nozzle, level 6), the plate was incubated again for 30 min at 37°C. Inhibitory zones appeared colorless (white) on a formazan-purple background. The positive control was tetracycline (10 μg/ml in ethanol, 0.4, 0.8, and 1.2 μl/band).

#### 2.4.2 *Aliivibrio fischeri* Bioassay

The bioluminescent marine Gram-negative bacteria *A. fischeri* were cultured according to DIN EN ISO 11348-1, Section 5 (European Committee for Standardization, 2009). Therefore, 200 µl of cryostock were suspended in 20 ml medium. The cultivation was performed overnight (18–24 h) in a 100 ml Erlenmeyer flask at room temperature by shaking at 75 rpm. Once the culture showed brilliant blue fluorescence by shaking in the dark, it was ready for use. An aliquot of the bacteria suspension (3 ml) was sprayed on the plate (blue nozzle, level 6) and directly recorded (BioLuminizer 2, CAMAG) (Morlock et al., 2021a; Morlock et al., 2021b). The native bioluminescence (depicted as a greyscale image) was documented in ten images at time intervals of 3 min. Exposure time was set to 120 s. Antibacterial components were detected as dark zones, whereas metabolism-enhancing substances appeared as bright zones on the bioluminescent background. The positive control was caffeine (1 mg/ml in methanol, 0.5, 1.5, and 3 µl/band).

#### 2.4.3 Cholinesterase Inhibition Assays

The initial AChE and BChE inhibition assays (Marston et al., 2002) were modified (Hage and Morlock, 2017; Morlock et al., 2021b). The plates were pre-wetted with 0.5 ml TRIS-HCl buffer (7.55 mg/ml TRIS, pH 7.8 adjusted with HCl, green nozzle, level 6). Then, 1.5 ml of enzyme solution (AChE 6.66 U/ml, BChE 3.34 U/ml, and each 1 mg/ml BSA in TRIS-HCl buffer) were applied (green nozzle, level 6) and the chromatogram was subsequently incubated at 37°C for 30 min. For detection, 0.5 ml substrate mixture (1 mg/ml 1-naphthyl acetate, 2 mg/ml Fast Blue B salt) was sprayed (red nozzle, level 6) onto the plate to obtain colorless (white) inhibition zones on a purple background. The positive control was rivastigmine (0.1 mg/ml in methanol, 2, 4, and 8 μl/band).

#### 2.4.4 Glucosidase Inhibition Assays

An improved version of Simões-Pires *et al.* (Simões-Pires et al., 2009) was used to detect α- and β-glucosidase inhibitors. The substrate solution (12 mg 2-naphthyl-α-d-glucopyranoside or 2-naphthyl-β-d-glucopyranoside in 9 ml ethanol and adding 1 ml of 10 mM NaCl solution) was sprayed (1 ml, red nozzle, level 6) onto the plate, followed by drying in a stream of cold air. Pre-wetting was carried out by spraying 0.5 ml sodium acetate buffer (41 mg/ml, pH 7.5 adjusted with 0.1 mM acetic acid, green nozzle, level 6). An aliquot of the respective enzyme solution (α-glucosidase 10 U/ml, β-glucosidase 1,000 U/ml in sodium acetate buffer) was applied (1 ml; green nozzle, level 6) and the plate was subsequently incubated at 37°C for 30 min. The antidiabetic effect was visualized by Fast Blue B salt staining (2 mg/ml in water, 0.5 ml, red nozzle, level 6), resulting in colorless (white) inhibitory zones on a purple background. The positive controls were acarbose (3 mg/ml in ethanol, 1, 3, and 6 μl/band) for the α-glucosidase assay and imidazole (1 mg/ml in ethanol, 3, 5, and 7 μl/band) for the β-glucosidase assay.

#### 2.4.5 β-Glucuronidase Inhibition Assay

The β-glucuronidase inhibition assay was run as described recently (Mahran et al., 2020) The chromatogram was pre-wetted with potassium phosphate buffer (0.5 ml; 9.34 mg/ml K_2_HPO_4_ and 6.31 mg/ml KH_2_PO_4_; green nozzle, level 6). Then, 750 µL enzyme solution (25 U/ml in potassium phosphate buffer with 1 mg/ml BSA) were sprayed onto the chromatogram (green nozzle, level 6). Incubation followed for 15 min at 37°C. As substrate, 750 µl of a 2 mg/ml 5-bromo-4-chloro-3-indolyl-β-d-glucuronide sodium salt solution was sprayed. The plate was incubated again for 60 min for producing colorless (white) inhibitory zones on a blue background. The positive control was d-saccharolactone (0.1 mg/ml in water, 1, 1.5, and 2 µl/band).

#### 2.4.6 Tyrosinase Inhibition Assay

The tyrosinase inhibitor potential was investigated according to an improved (Morlock et al., 2021b) workflow (Taibon et al., 2015). To prepare the substrate solution, 45 mg levodopa, 25 mg CHAPS, and 75 mg PEG 8000 were dissolved in 10 ml of phosphate buffer (1.4 mg/ml K_2_HPO_4_, 1.68 mg/ml Na_2_HPO_4_, pH 6.8) and stored at 4°C until use. The levodopa substrate solution was sprayed onto the chromatogram (1 ml, blue nozzle, level 6) and subsequently dried for 2 min in a stream of cold air. Then, 1 ml of enzyme solution (400 U/ml in phosphate buffer) was sprayed onto the plate (blue nozzle, level 6), followed by incubation at room temperature for 20 min. After incubation, the plate was immediately dried and documented. Tyrosinase inhibition activity was apparent as colorless (white) zones on a greyish-brown background. The positive control was kojic acid (0.1 mg/ml in ethanol, 1, 3, and 6 μl/band).

#### 2.4.7 Planar Yeast Androgen/Estrogen Screen (pYAS/pYES) Bioassay

The hormonal-effective bioassays were run as recently described (Klingelhöfer et al., 2020). Cryogenic YAS or YES cell culture (1 ml each) was suspended in 39 ml or 29 ml medium, respectively. The suspensions were cultivated by shaking at 70–75 rpm and 30°C overnight (20–22 h). The cell number was determined with a hemocytometer after diluting 50 µl culture in 950 µl 0.9% NaCl solution. The required cell count of 0.8 × 10^8^ cells/ml was adjusted via centrifugation (2,500 × *g*, 5 min) of 5 ml yeast cell culture and resuspension in the required amount of medium plus 50 µl copper sulfate. This suspension was sprayed on the plate (1.4 ml, red nozzle, level 6), followed by incubation for 4 h (pYAS) or 3 h (pYES) at 30°C. Substrate solution (2 mg 4-methylumbelliferyl-β-d-galactopyranoside, 100 µl DMSO, 3 ml citrate buffer) was sprayed onto the chromatogram (1.5 ml, yellow ultra-nozzle, level 2). Subsequently, the plates were incubated for 1 h at 37°C. Bioautograms were recorded at FLD 366 nm. Endocrine agonists appeared as 4-methylumbelliferone-blue fluorescent zones on a dark blue background. As a positive control, testosterone (for pYAS: 0.5 µL, 1.5 μg/ml in methanol) or 17-β-estradiol (for pYES: 5 μl, 100 ng/ml in ethanol) were applied.

#### 2.4.8 Metabolization via S9–pYES Bioassay

Potential estrogens resulting from liver metabolism were investigated by adding the S9 enzyme mixture (500 µl) and respective cofactors (166 µl NADP, 42 µl glucose 6-phosphate, 958 µl phosphate buffer) to 3,334 µl *Saccharomyces cerevisiae* cell culture (0.8 × 10^8^ cells/ml). The assay was performed as described above.

#### 2.4.9 Planar Yeast Antagonistic Androgen/Estrogen Screen (pYAS/pYES) Bioassay

To screen the antagonistic activity, the pYAS or pYES bioassays were extended by overspraying along the middle of each track a 1 mm × 70 mm area of testosterone (4 μl, 1.5 μg/ml in methanol) or 17-β-estradiol (5 μl, 2 ng/ml in ethanol), respectively, with the Freemode option of winCATS (Klingelhöfer et al., 2020). Endocrine antagonists appeared as fluorescence-reducing bands in the 4-methylumbelliferone-blue fluorescent testo-sterone or 17-β-estradiol track part.

#### 2.4.10 SOS-Umu-C Bioassay

The planar SOS-Umu-C bioassay was run on HPTLC silica gel plates without a fluorescence indicator. After development, the plates were additionally scanned at 546/>580 nm using the TLC Scanner 3 (CAMAG). *Salmonella typhimurium* cells (50 µl cryostock) were suspended in 35 ml Lysogeny broth (20 mg/ml with 1 mg/ml d-(+)-glucose and 106 mg/L ampicillin sodium salt) and incubated overnight at 75 rpm and 37°C for 16 h. Before use, the cells were centrifuged (3,000 × *g*, 10 min). The pelleted cells were re-suspended in fresh medium to obtain the required OD_660_ of 0.2 (Meyer et al., 2020). The chromatogram was sprayed with *Salmonella* suspension (2.8 ml, yellow nozzle, level 3) and incubated at 37°C for 3 h. Substrate solution [15 µl resorufin-β-d-galactopyranoside solution (20 mg/ml in DMSO) in 2.1 ml phosphate buffer and 0.9 ml glycerol] was sprayed onto the plate (2.5 ml, red nozzle, level 6). Incubation followed at 37°C for 1 h. The plates were documented at white light illumination and 366 nm. The generated resorufin fluorescence was measured at 546/>580 nm. Genotoxic substances were detectable either as pink zones on the colorless background at white light illumination or as pink fluorescent zones on a brown-reddish background at 254 nm or 366 nm. The positive control was 4-nitroquinoline-1-oxide (1 μg/ml in methanol, 1 µl/spot).

#### 2.4.11 α-Amylase Inhibition Assay

The latest α-amylase inhibition method, which used immersion of the plate into enzyme and substrate solutions (Agatonovic-Kustrin and Morton, 2017; Agatonovic-Kustrin et al., 2019), was adjusted and transferred to a piezoelectric spraying procedure, in which the enzyme solution (62.5 U/mL in sodium acetate buffer) was sprayed onto the chromatogram (1 ml, red nozzle, level 5), followed by 30 min incubation at 37°C. As substrate 2%-soluble starch solution was sprayed onto the wet plate (0.5 ml, red nozzle, level 5). After 20 min incubation at 37°C, Gram’s iodine solution was sprayed (250 μl, yellow nozzle, level 5) for visualization. The α-amylase inhibition activity was observed as violet zones on a colorless background. The positive control was acarbose (0.1 mg/ml in methanol, 0.3, 0.6, and 0.9 µl/band).

### 2.5 Instrumental Setup of the 8D-Hyphenation

The multipotent bioactive zones were further characterized with RP-HPLC-DAD-HESI-MS directly after EDA. The UPLC system (Acquity H Class, Waters, Eschborn, Germany) was equipped with the quaternary solvent manager, solvent degasser, sample manager, column oven, photodiode array detector (DAD), and HESI-MS (single quadrupole QDa, Waters). The bioactive target zone was heart-cut eluted with an oval elution head (4 mm × 2 mm) of the TLC-MS Interface 2 (CAMAG) with 90% aqueous methanol. A standalone pump supplied the solvent (515 HPLC pump, Waters). Analytes were transferred through a biocompatible inline filter (IDEX Health and Science, Oak Harbor, WA, United States) containing a PEEK frit (0.5 µm, Techlab, Brunswick, Germany) to an online desalting RP pre-column/defender guard (Accucore RP-MS, 10 mm × 2.1 mm, 2.6 µm, Thermo Scientific, Bellefonte, PA, United States). The online desalting device was installed onto a two-position switching valve (MXT-Series PD715-000, Rheodyne IDEX Health and Science) and served as an analyte trap while discarding the bioassay salts as waste. By switching, controlled via remote control and Rheodyne TitanMX software, the analytes were transferred to the main RP column (Accucore RP-MS 100 mm × 2.1 mm, 2.6 µm, Thermo Scientific) and separated orthogonally. The column was thermostated at 40°C. The 13 min HPLC gradient consisted of (A) 2.5 mM ammonium acetate (pH 4.5 adjusted with acetic acid) and (B) methanol. Starting conditions were 98% A at a flow rate of 0.6 ml/min for the first 2 min. The methanolic portion increased linearly to 20% within the following 2.5 min. At 8 min, a ratio of 10/90% A/B was reached and held for the next 2 min; then it fell to 98% A within 0.1 min, followed by 3 min equilibration time. Detection parameters were set to a wavelength scan from 190 to 400 nm for DAD. The MS was operated in polarity-switching mode, while the ESI probe was heated to 600°C and ESI source to 120°C. The sampling frequency was set to 5 Hz and cone voltage to ±10 V in both ionization modes (Schreiner and Morlock, 2021). The MassLynx V4.2 software (Waters) was used to evaluate and process the data.

## 3 Results and Discussion

### 3.1 Outline of the Study

**FIGURE 1:**
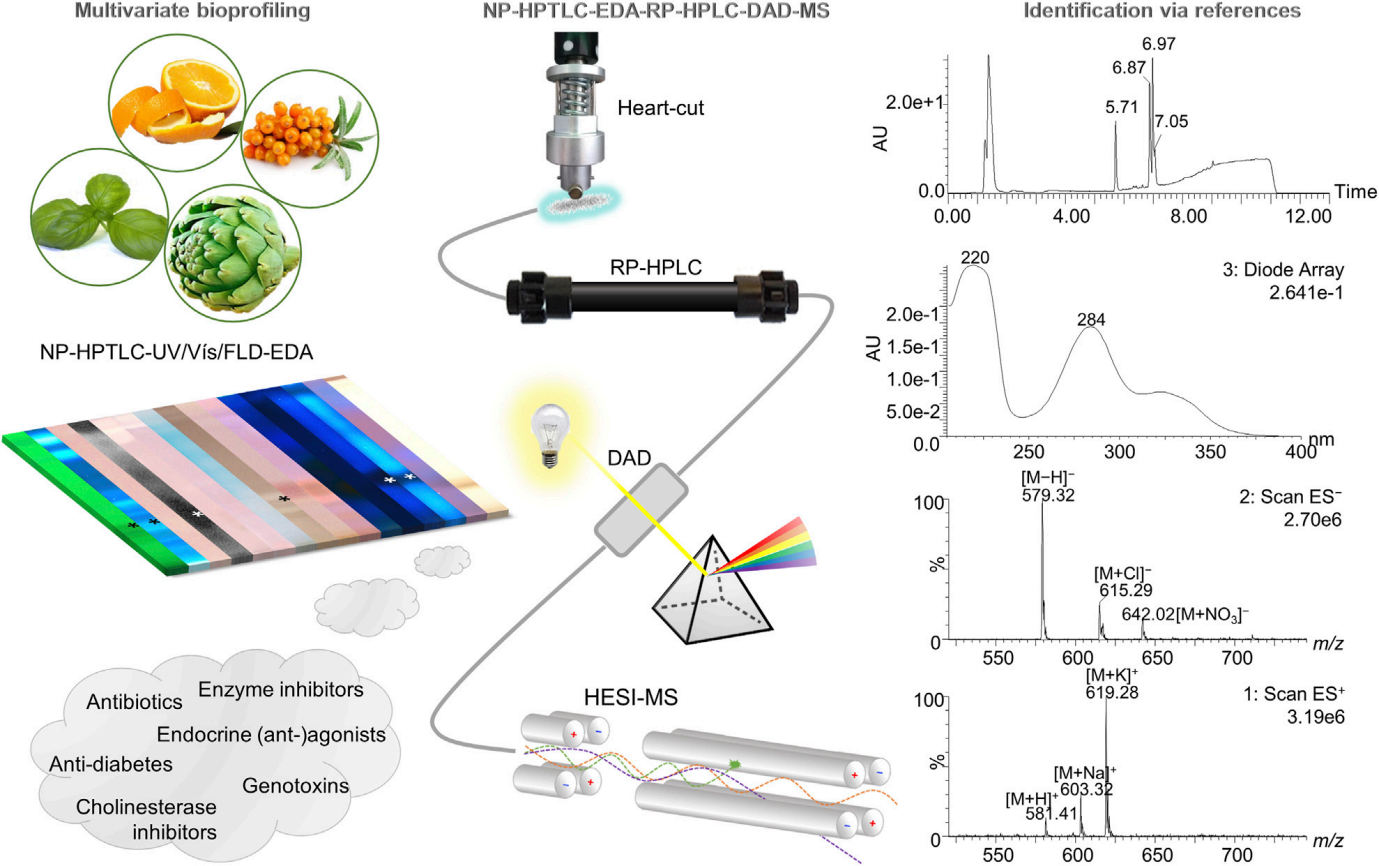
Schematic overview of the hyphenation NP-HPTLC-UV/Vis/FLD-EDA-heart-cut RP-HPLC-DAD-HESI-MS. Botanical extracts were separated on NP-HPTLC plates followed by biochemical and microbiological detections. Bioactive zones were heart-cut eluted directly out of the (bio)autogram and transferred to the orthogonal RP-HPLC-DAD-HESI-MS, resulting in comprehensive information about a distinct bioactive compound.

**FIGURE 2:**
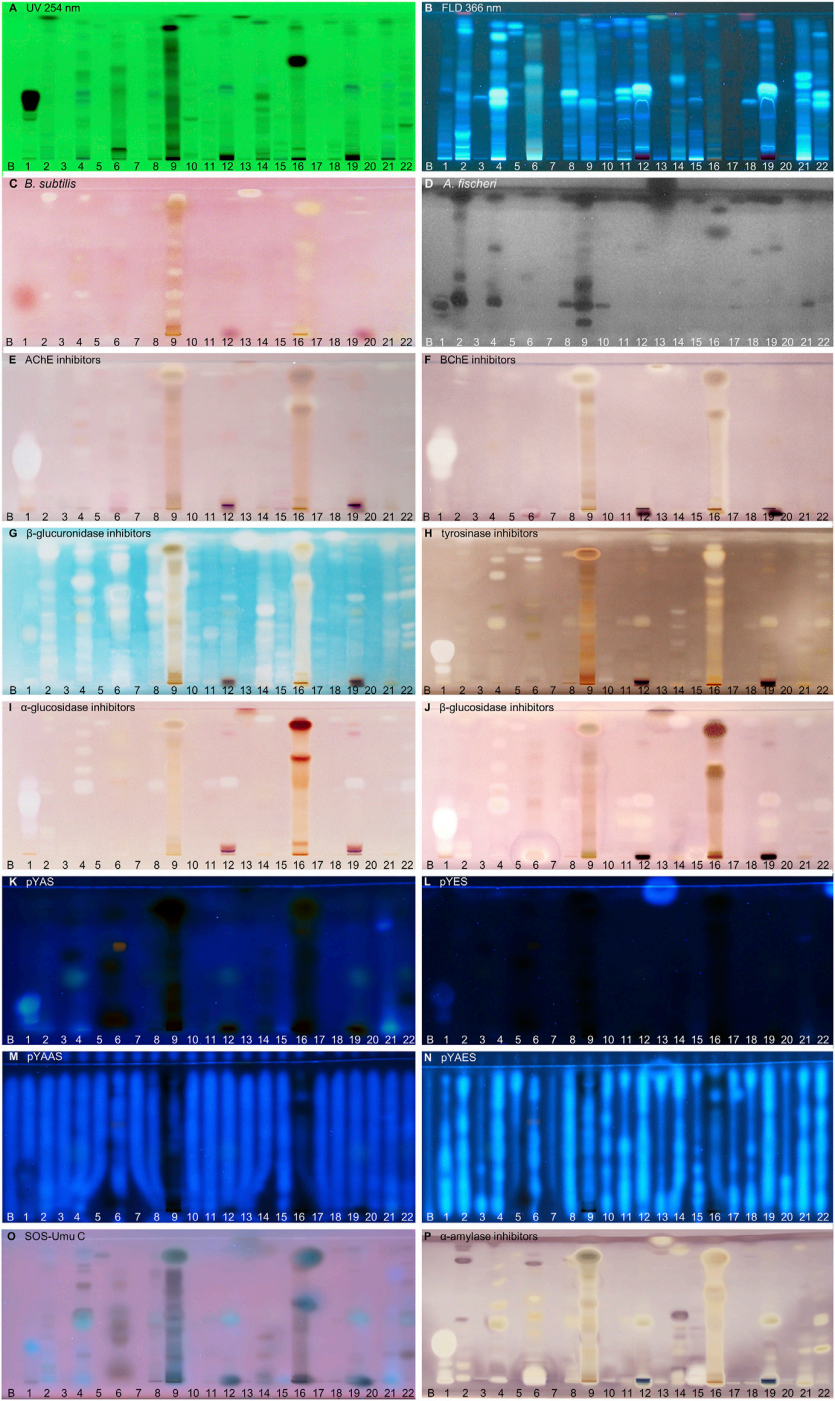
NP-HPTLC−UV/Vis/FLD−EDA profiles of the plant extracts no. 1–22. Separation of the applied botanicals (4 µL/band, assignments in Table 1; solvent blank B for comparison) on HPTLC plate silica gel 60 F_254_ MS-grade with ethyl acetate—toluene—formic acid—water (16:4:3:2, *V/V/V/V*) up to 70 mm, detected at UV 254 nm **(A)**, FLD 366 nm **(B, K–O)** and white light illumination **(C, E–J, P)** after the *B. subtilis* bioassay **(C)**, *A. fischeri* bioassay with bioluminescence depicted as a greyscale image, **(D)** and AChE **(E)**, BChE **(F)**, β-glucuronidase **(G)**, tyrosinase **(H)**, α-glucosidase **(I)**, β-glucosidase **(J)** and α-amylase **(P)** inhibition assays, as well as pYAS **(K)**, pYES **(L)**, pYAAS **(M)**, pYAES **(N)**, SOS-Umu-C **(O)** bioassays.

**FIGURE 3:**
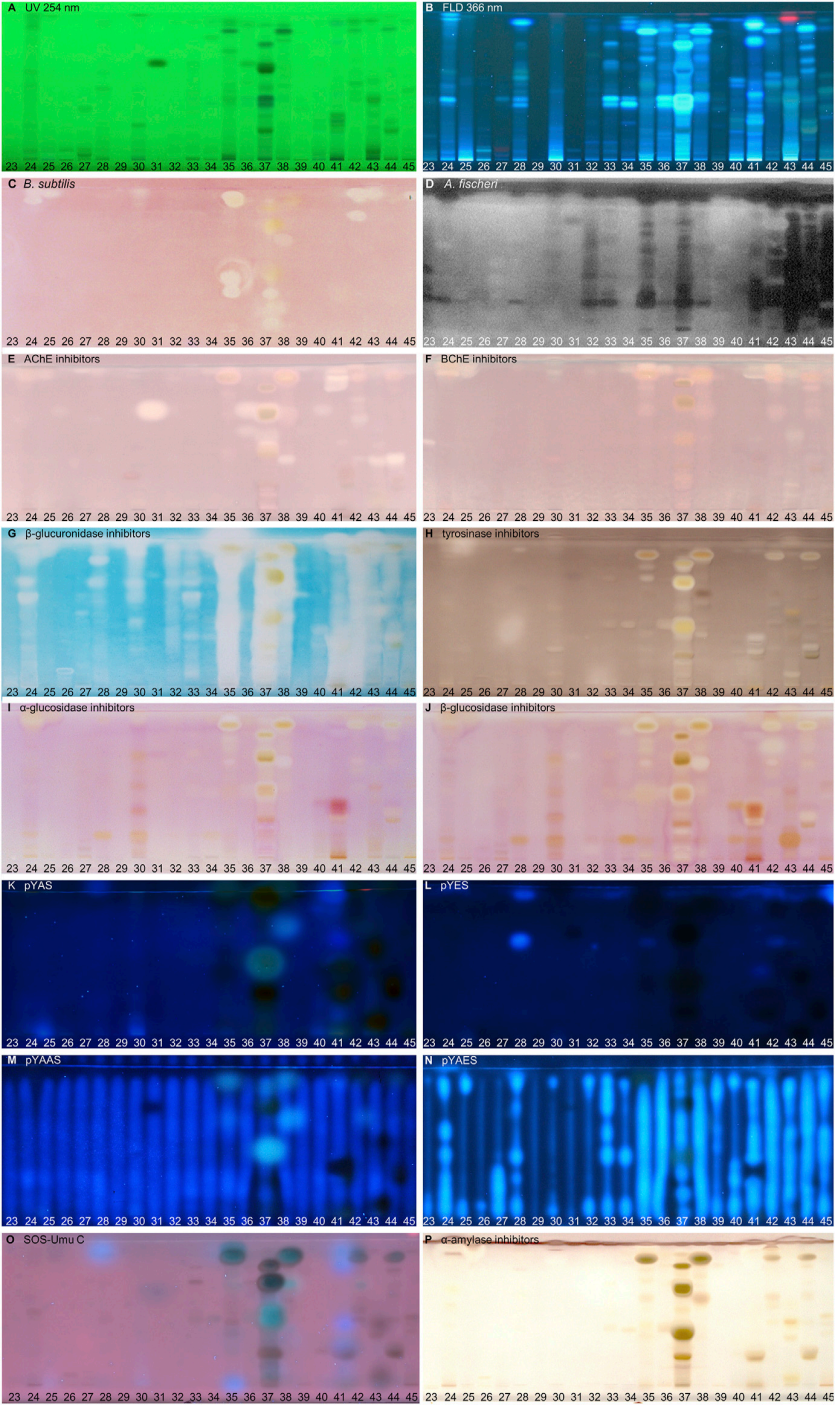
NP-HPTLC−UV/Vis/FLD−EDA profiles of the plant extracts no. 23–45. Separation of the applied botanicals (4 µL/band, assignments in Table 1) on HPTLC plate silica gel 60 F_254_ MS-grade with ethyl acetate—toluene—formic acid—water (16:4:3:2, *V/V/V/V*) up to 70 mm, detected at UV 254 nm **(A)**, FLD 366 nm **(B, K–O)** and white light illumination **(C, E–J, P)** after the *B. subtilis* bioassay **(C)**, *A. fischeri* bioassay with bioluminescence depicted as a greyscale image, **(D)** and AChE **(E)**, BChE **(F)**, β-glucuronidase **(G)**, tyrosinase **(H)**, α-glucosidase **(I)**, β-glucosidase **(J)**, and α-amylase **(P)** inhibition assays, as well as pYAS **(K)**, pYES **(L)**, pYAAS **(M)**, pYAES **(N)**, SOS-Umu-C **(O)** bioassays.

**FIGURE 4:**
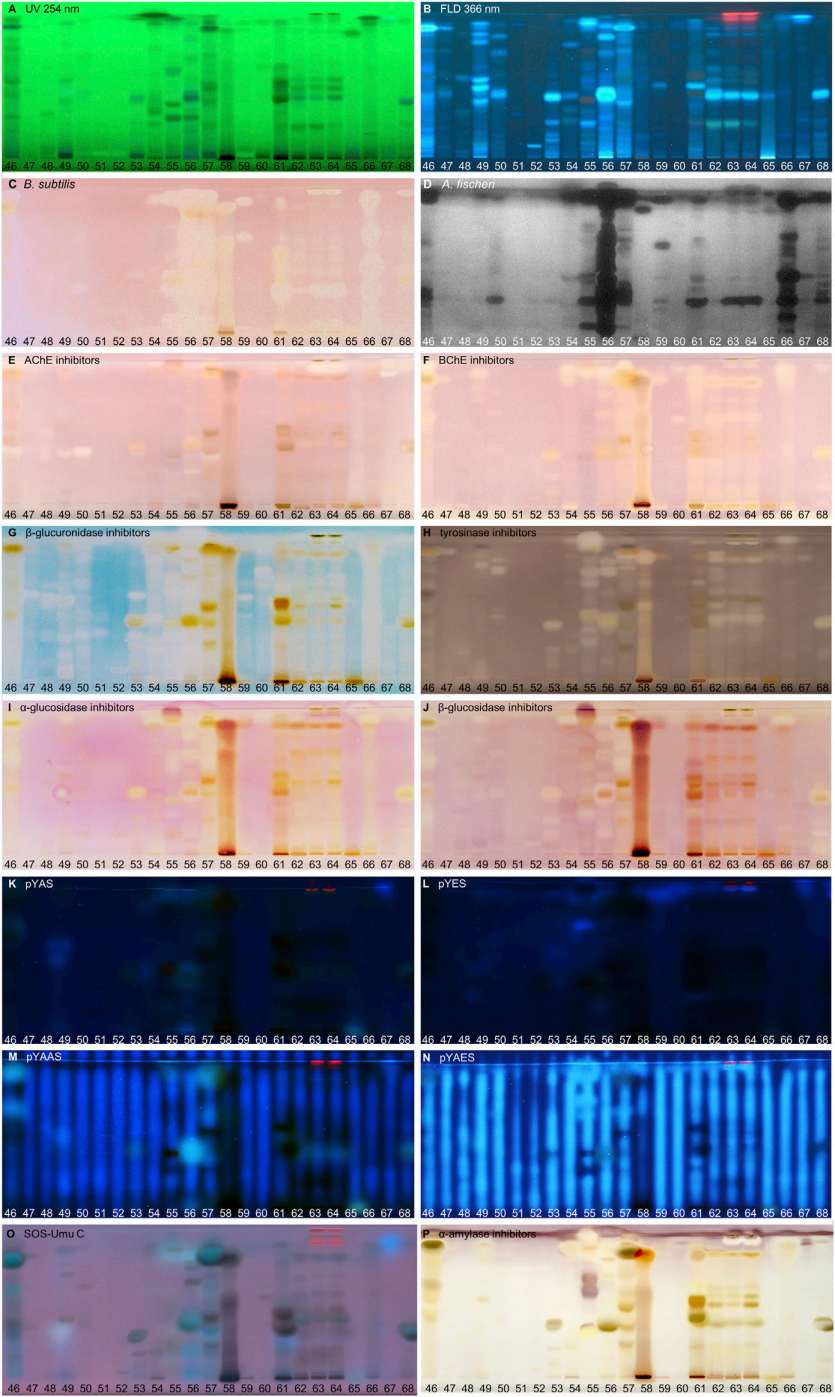
NP-HPTLC−UV/Vis/FLD−EDA profiles of the plant extracts no. 46–68. Separation of the applied botanicals (4 µL/band, assignments in Table 1; solvent blank B for comparison) on HPTLC plate silica gel 60 F_254_ MS-grade with ethyl acetate—toluene—formic acid—water (16:4:3:2, *V/V/V/V*) up to 70 mm, detected at UV 254 nm **(A)**, FLD 366 nm **(B, K–O)**, and white light illumination **(C, E–J, P)** after the *B. subtilis* bioassay **(C)**, *A. fischeri* bioassay with bioluminescence depicted as a greyscale image, **(D)** and AChE **(E)**, BChE **(F)**, β-glucuronidase **(G)**, tyrosinase **(H)**, α-glucosidase **(I)**, β-glucosidase **(J)**, and α-amylase **(P)** inhibition assays, as well as pYAS **(K)**, pYES **(L)**, pYAAS **(M)**, pYAES **(N)**, SOS-Umu-C **(O)** bioassays.

**FIGURE 5:**
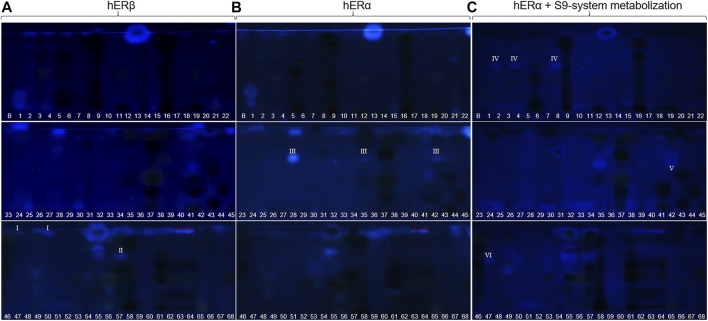
Comparison and observed differences (I−VI) of the estrogenic activity via NP-HPTLC–pYES using the hERα versus hERβ used for the first time, or after metabolization with the S9 mixture. Separation of the applied botanicals (no. 1–68, 4 µl/band, assignments in Table 1; solvent blank B for comparison) on HPTLC plate silica gel 60 F_254_ MS-grade with ethyl acetate—toluene—formic acid—water (16:4:3:2, *V/V/V/V*) up to 70 mm, detected at FLD 366 nm after pYES. Cells were equipped either with hERβ **(A)**, hERα **(B)** or phytochemicals were metabolized with the S9 enzyme mixture, simulating liver metabolism **(C)**.

**FIGURE 6:**
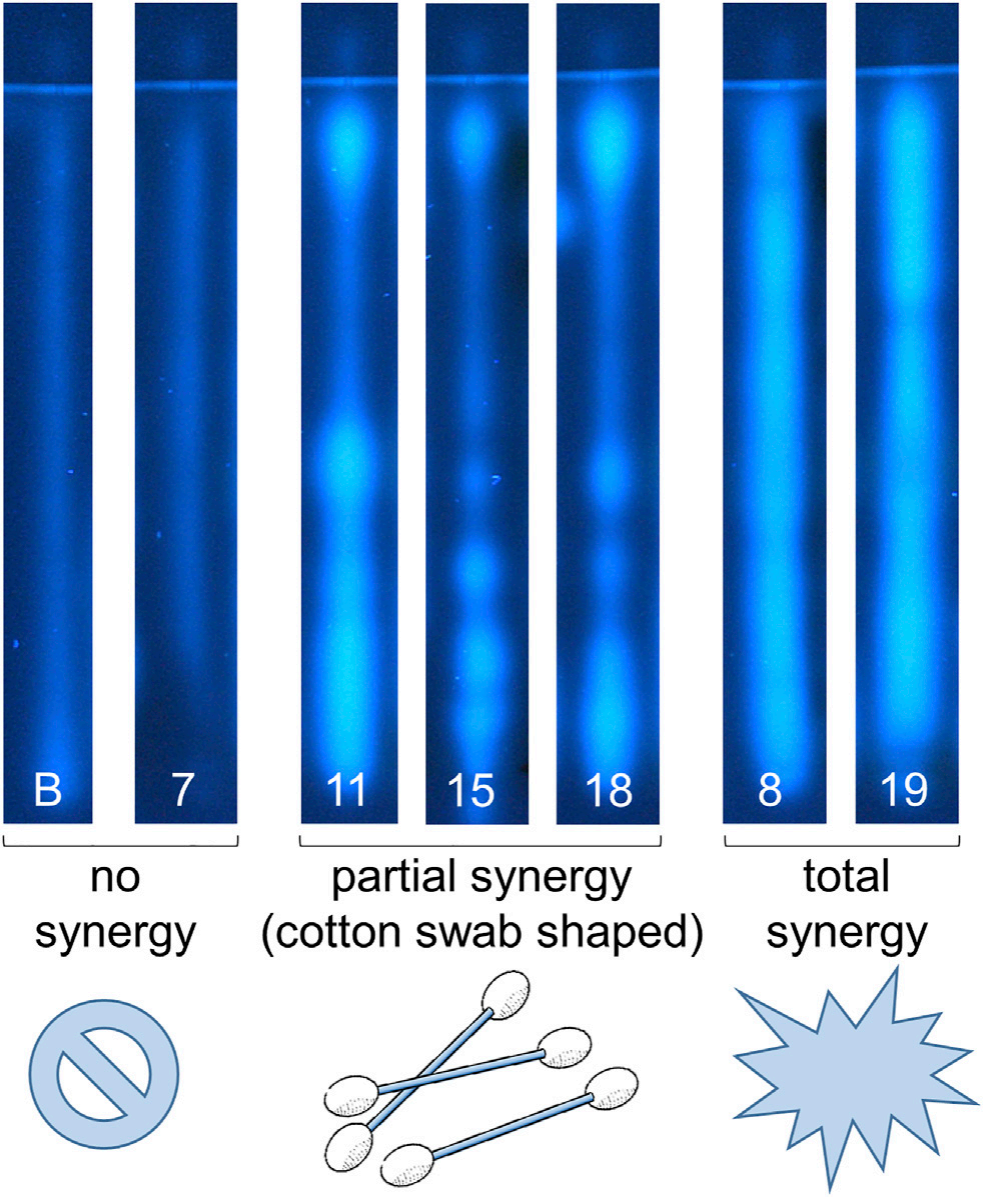
First report of synergy profiles, exemplarily shown for the NP-HPTLC-pYAES bioassay. Separation of the applied botanicals (nos. 7–19, assigned in Table 1; 4 µl/band each; solvent blank B for comparison) on HPTLC plate silica gel 60 F_254_ MS-grade with ethyl acetate—toluene—formic acid—water (16:4:3:2, *V/V/V/V*) up to 70 mm; each separated track was oversprayed by an 1 mm × 70 mm area of 17-β-estradiol (10 ng/band) and after bioassay application detected at FLD 366 nm. Fortified estrogenic fluorescence was interpreted as synergistic effects, which were not observed in solvent blank B and no. 7 (only a slight anti-estrogenic effect in the lower *hR*_F_ range), partially observed as cotton-swab-shaped 17-β-estradiol band (nos. 11, 15 and 18) or as overall broadened estrogenic band (nos. 8 and 19), if compared to the solvent blank B, which shows reference 17-β-estradiol fluorescence.

**FIGURE 7:**
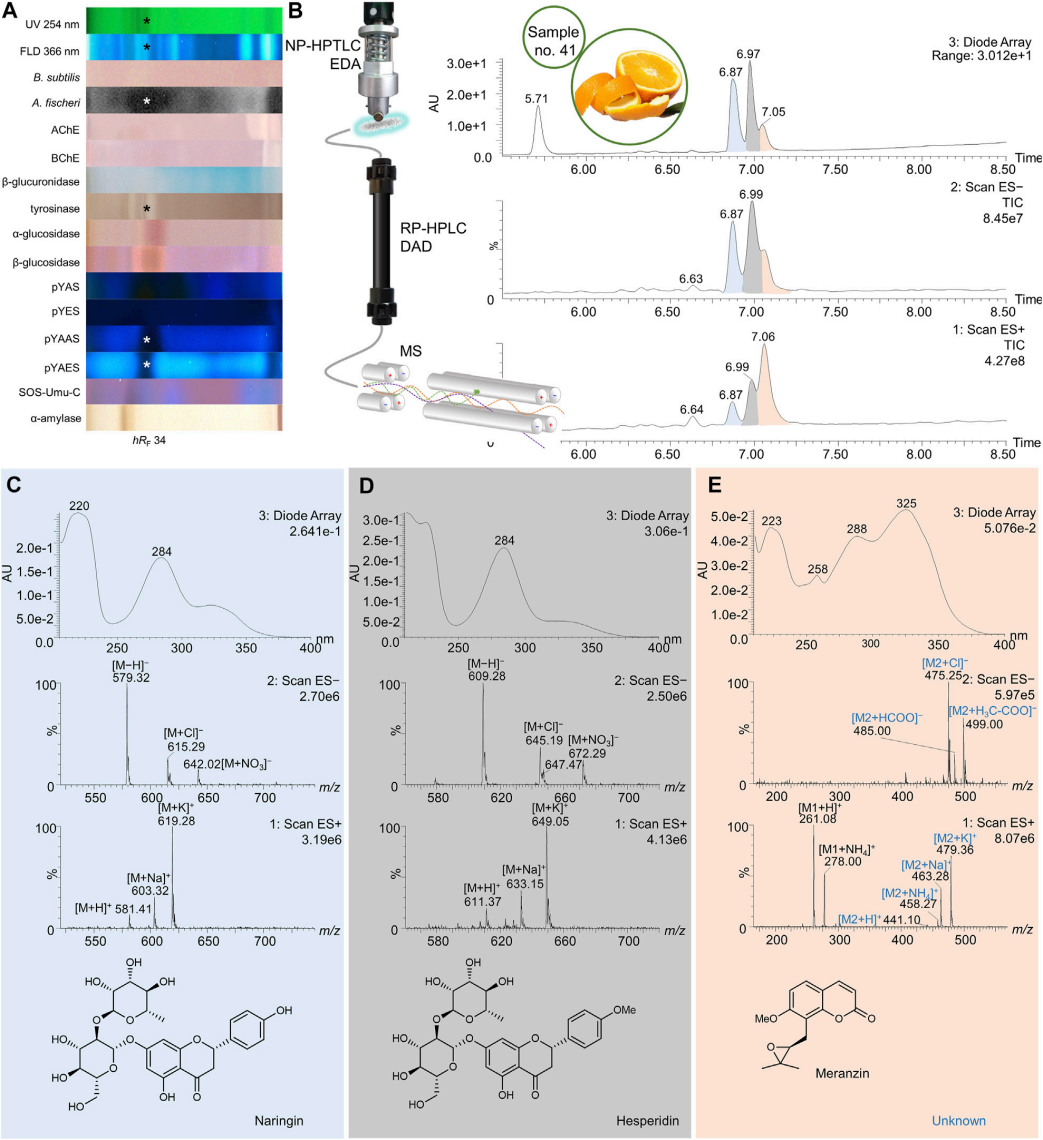
NP-HPTLC−UV/Vis/FLD−EDA−heart-cut RP-HPLC−DAD−HESI-MS analysis of orange peel no. 41. NP-HPTLC−UV/Vis/FLD−EDA analysis as in Figure 3 **(A)**, followed by heart-cut elution of the bioactive zone at hR_F_ 34 (marked*) directly out of the pYAES bioautogram and online desalting to obtain the RP-HPLC−DAD−HESI-MS chromatograms **(B)** and respective UV and mass spectra of the peaks at RT 6.87 min **(C)**, 6.98 min **(D)**, and 7.05 min **(E)**.

**FIGURE 8:**
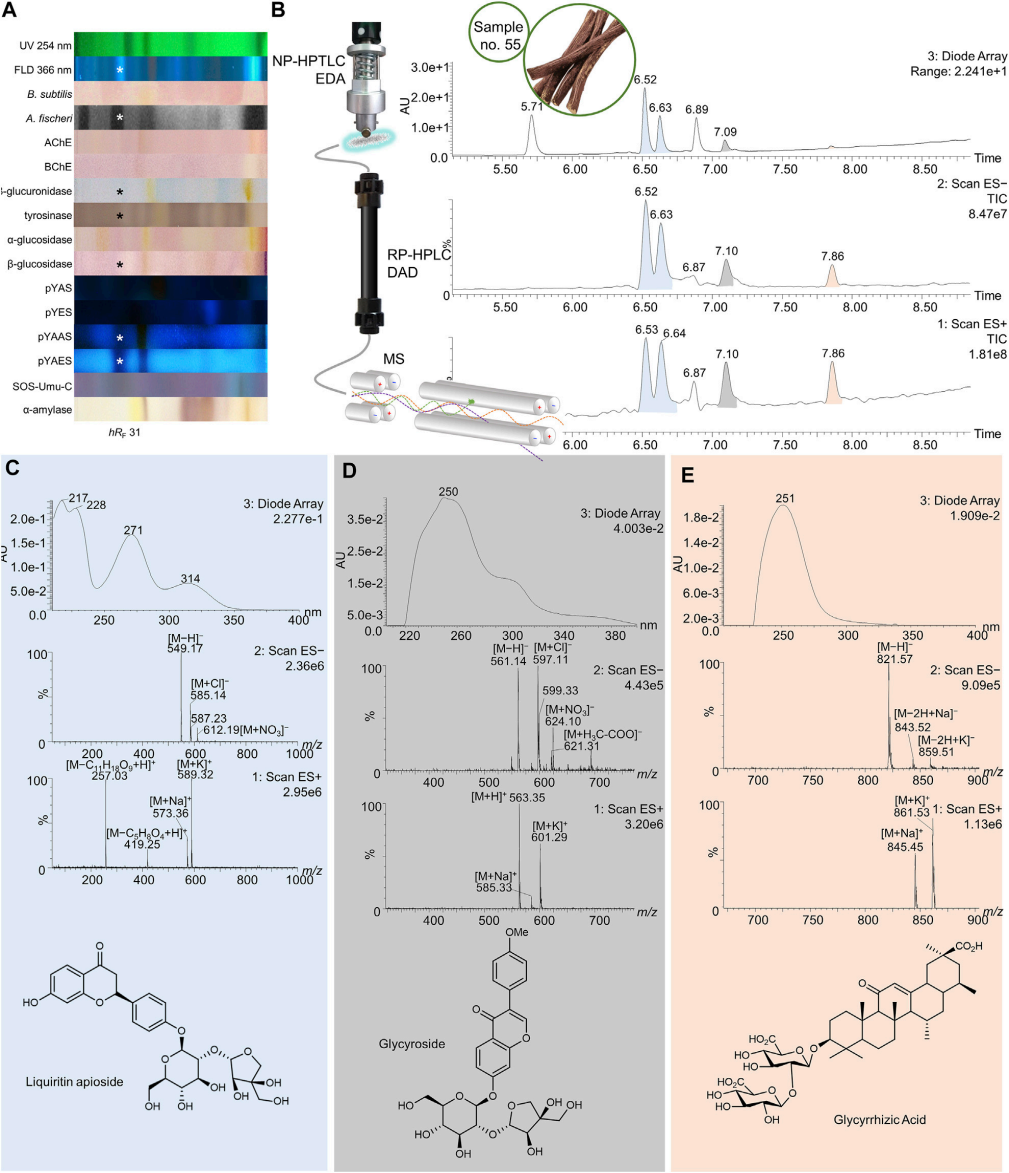
NP-HPTLC−UV/Vis/FLD−EDA−heart-cut RP-HPLC−DAD−HESI-MS analysis of licorice no. 55. NP-HPTLC−UV/Vis/FLD−EDA analysis as in Figure 4 **(A)**, followed by heart-cut elution of the bioactive zone at hR_F_ 31 (marked*) directly out of the pYAES bioautogram and online desalting to obtain the RP-HPLC−DAD−HESI-MS chromatograms **(B)** and respective UV and mass spectra of the peaks at RT 6.53/6.64 min **(C)**, 7.10 min **(D)**, and 7.86 min **(E)**.

**FIGURE 9:**
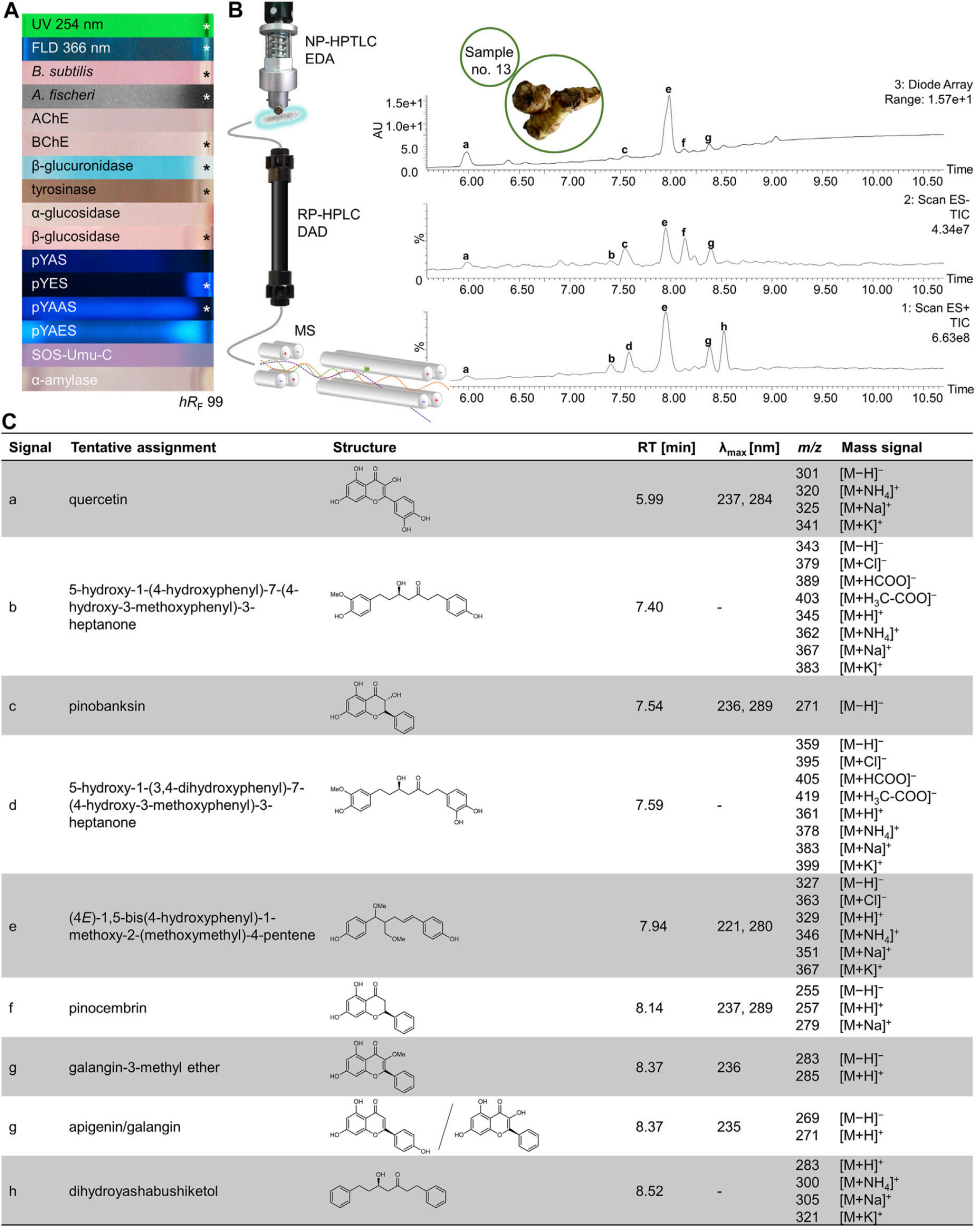
NP-HPTLC−UV/Vis/FLD−EDA−heart-cut RP-HPLC−DAD−HESI-MS analysis of galangal no. 13. NP-HPTLC−UV/Vis/FLD−EDA analysis as in Figure 2 **(A)**, followed by heart-cut elution of the bioactive zone at hR_F_ 99 (marked*) directly out of the BChE autogram and online desalting to obtain the RP-HPLC−DAD−HESI-MS chromatograms **(B)** and respective UV and mass spectral data of the peaks a−h **(C)**.

**FIGURE 10:**
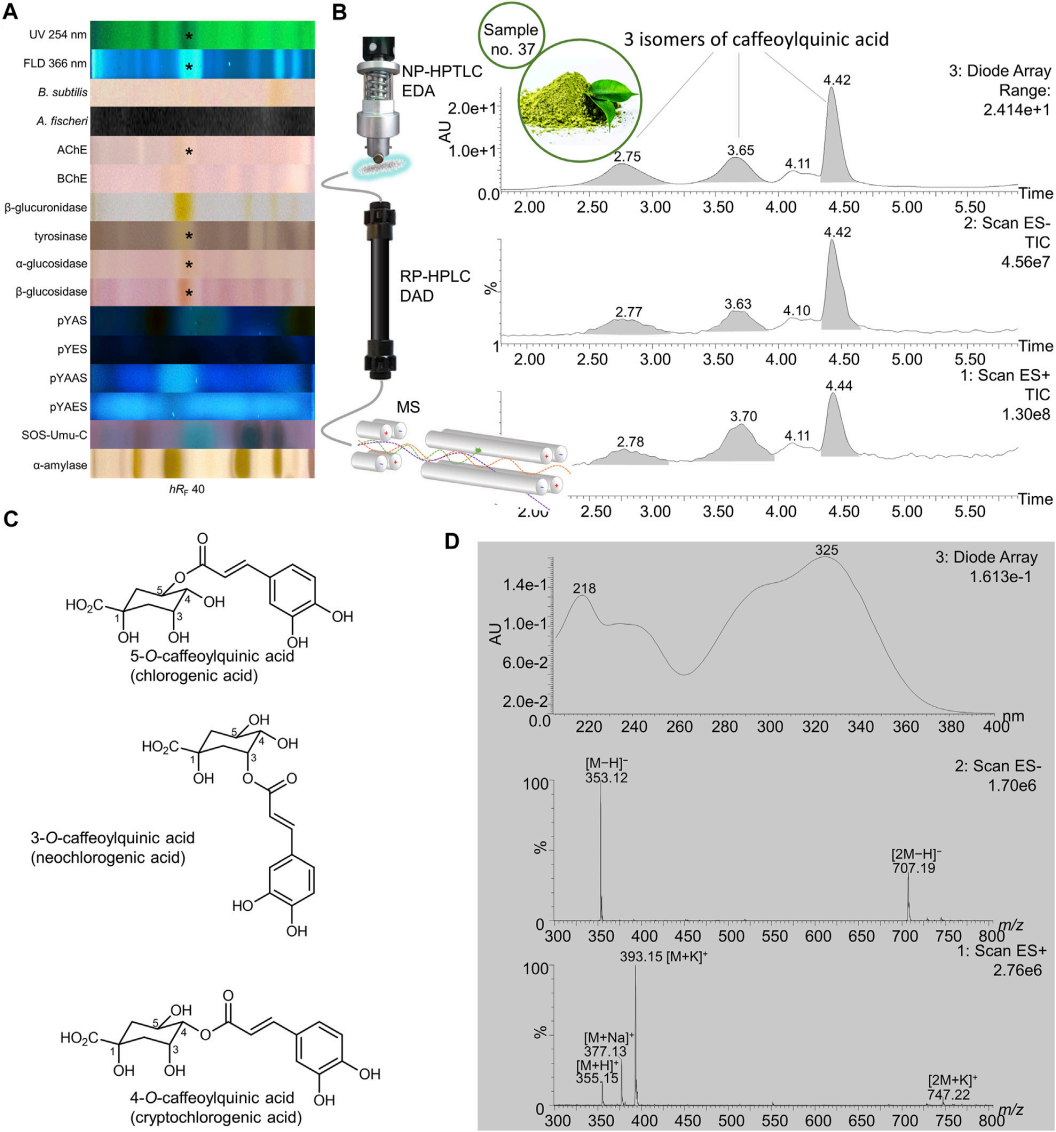
NP-HPTLC−UV/Vis/FLD−EDA−heart-cut RP-HPLC−DAD−HESI-MS analysis of yerba mate green no. 37. NP-HPTLC−UV/Vis/FLD−EDA analysis as in Figure 3 **(A)**, followed by heart-cut elution of the bioactive zone at hR_F_ 40 (marked*) directly out of the BChE autogram and online desalting to obtain the RP-HPLC−DAD−HESI-MS chromatograms **(B)** and respective UV and mass spectra for the peaks at RT 2.78, 3.70, and 4.44 min, which were assigned to the isomeric structures of caffeoylquinic acid with the most predominant depicted, **(C)** as all three peaks provided the same signals **(D)**.

**FIGURE 11:**
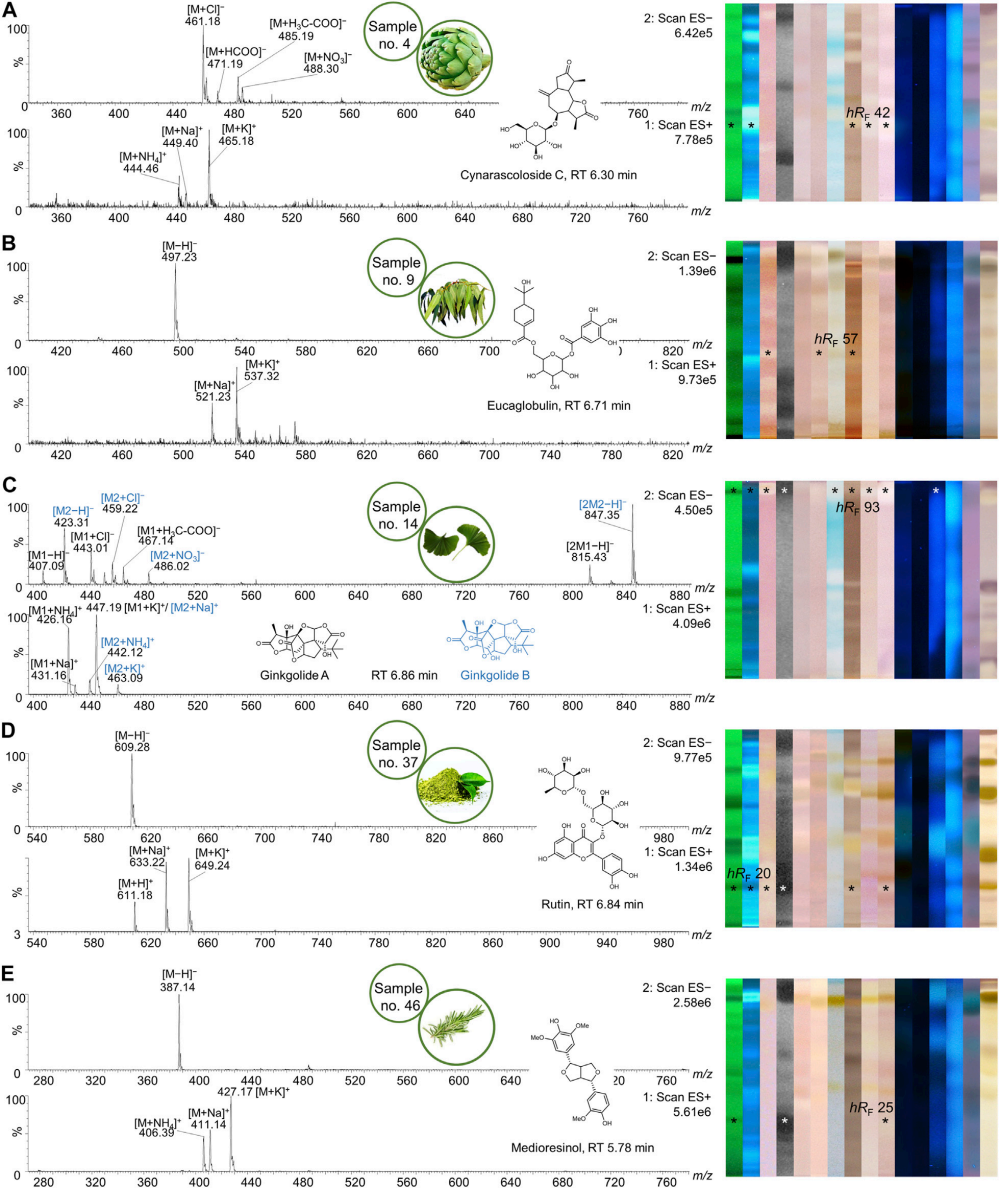
Mass spectra of multipotent bioactive zones. NP-HPTLC−UV/Vis/FLD−EDA analysis as in Figures 2–4, followed by heart-cut elution of the multipotent bioactive zones of artichoke **(A)**, eucalyptus **(B)**, ginkgo **(C)**, yerba mate green **(D)**, and rosemary **(E)**. Pure mass spectra of respective HPLC peaks are shown with their associated structural formula and respective HPTLC fingerprints (bioactive zones marked*).

A total of 68 very different powdered plant extracts (botanicals added to food products in food industry) and 16 different effect-directed assays were selected to investigate and prove the suitability of the biological–physicochemical 8D hyphenation for generic screening (Figure 1). Among the plants (Table 1) were such ones that are commonly used as culinary spices and herbs or in traditional medicine. Their diverse and varying compositions represent different matrix loads for the analytical system. Moreover, the nine biological and seven biochemical assay media differed over a wide range in salt and nutrient composition. This represents the diversity of possible compositions of a bioactive zone (to be heart-cut and transferred to the next dimension) and was therefore considered a good worst-case scenario to test whether the developed generic hyphenation method is suitable for routine analyses. First, the bioactivity screening was evaluated per assay (Figures 2–6), whereby some botanicals were mentioned repeatedly, *i.e.* galangal (no. 13) yerba mate (no. 37), orange peel (no. 41), licorice (no. 55), and Siberian ginseng (no. 56). Then, these botanicals were subjected to heart-cut RP-HPLC−DAD−HESI-MS analysis (Figures 7–11). All botanicals were extracted and applied analogously. Thus, the effect profiles of each assay could directly be compared by their response pattern. The most effective and important botanicals were highlighted at a glance in side-by-side comparison. The band broadening (diffusion) depended on the assay incubation time. The most important bioactive compounds discovered were tentatively assigned based on information obtained about spectral (UV/Vis/FLD), polarity (*hR*_F_ values with a deviation of ±1), and molecular (mass signal) properties. Since there was no access to a high-resolution mass spectrometry system, the assignments were verified by comparing with reference standards.

Using the Gram-negative *Aliivibrio fischeri* and Gram-positive *Bacillus subtilis* bioassays, natural antibacterial compounds were detected that can subtly fight infections and contribute to longer shelf life and better preservation of products. Natural AChE and BChE inhibitors, which can provide symptomatic benefits for the cognitive decline of Alzheimer’s patients (Tundis et al., 2016), were revealed by the respective planar enzyme inhibition assays. The tyrosinase inhibition assay was used to screen for plant-based skin whiteners or inhibitors of the enzymatic food browning. The β-glucuronidase inhibition assay was used to detect compounds that prevent the gut-bacterial reversion of detoxification via glucuronidation. Additional α-amylase, α- and β-glucosidase inhibition assays were employed to determine natural compounds with benefits for diabetes patients. Phytoandrogens and phytoestrogens were detected via the human estrogen receptor α (hERα) or hERβ or in combination with the S9 enzyme mixture simulating liver metabolization, and respective antagonists were investigated using recombinant yeast cells equipped with the human estrogen/androgen receptor. Genotoxins were detected with a recombinant *Salmonella typhimurium* strain equipped with the SOS-Umu-C repair mechanism. Hence, the spectrum of effects in the investigated botanicals may be linked to the mitigation of bacterial infects, the improvement of cholinergic transmission for Alzheimer’s patients, the decrease of blood glucose levels in diabetics, the reduction of skin abnormalities, and to the balance of the steroid hormonal system, among other disorders.

### 3.2 Screening Results

#### 3.2.1 Compounds Inhibiting Bacteria

In traditional medicines, plant-based extracts are used to assist in the treatment of bacterial infections (Brantner and Grein, 1994; Palombo and Semple, 2001). Antibacterial activities can be so effective that plant extracts are also used as preservatives in food products. For example, the ingredients carnosol and carnosic acid of rosemary extract are marketed as preservative E 392. Rosemary (no. 46) is also screened here, using non-pathogenic bacterial representatives which are easier to handle in the laboratory. The Gram-positive *B. subtilis* bioassay is based on an oxidoreductase enzyme reaction. Intact enzymes of viable *B. subtilis* cells reduce the tetrazolium salt MTT to the insoluble purple formazan (Marston, 2011). Cell death is visualized as colorless zones indicating antibacterial compounds. Most antibacterials detected were located at *hR*_F_ values ≥ 90 (Figures 2–4C). In eucalyptus (no. 9), marjoram (no. 35), yerba mate green (no. 37), Siberian ginseng (no. 56), thyme (no. 57), hawthorn leaves (nos. 63 and 64), and cinnamon bark (no. 66) additional antibacterials were detected in the lower *hR*_F_ range. Essential oils from herbs such as oregano and thyme are known for their antimicrobial activity, especially against Gram-positive bacteria (Soković et al., 2010).

The Gram-negative *A. fischeri* bacteria are able to convert metabolic energy into turquoise bioluminescence via luciferase. A change in bioluminescence intensity is correlated with substances enhancing or reducing the cell metabolism. Such effects are visualized as lightened or dark zones on the bioluminescent background of the bioautogram (depicted as greyscale image). Almost all botanical extracts showed antimicrobial activity against *A. fischeri* (Figures 2–4D). Intense antimicrobial zones were detected in yerba mate green (no. 37), passionflower (no. 43), peppermint (no. 44), rooibos (no. 45), licorice (no. 55), Siberian ginseng (no. 56), and cinnamon bark (no. 66). Most samples showed at least one dark antimicrobial zone, while more than half of all samples had two or more. The more universally and sensitively detecting *A. fischeri* bioassay proved to be a good starting assay to investigate complex mixtures.

#### 3.2.2 Compounds Inhibiting AChE and BChE

In traditional or ayurvedic medicine, plants and their phytoconstituents are used to assist in the treatment of Alzheimer´s disease (Azadniya et al., 2021). The pathophysiology of Alzheimer’s disease is often associated with cholinergic system dysfunction. Therefore, many synthetic drugs target the inhibition of cholinesterases. Both AChE and BChE catalyze the hydrolysis of the neurotransmitter acetylcholine into acetic acid and choline. While AChE is highly selective for acetylcholine, BChE can also convert other substrates, *e.g.,* butyrylcholine, succinylcholine, or organophosphates. Besides synthetic drugs, also natural compounds are able to inhibit this enzyme mechanism. Particularly polyphenols interact with amino acid residues of the active side of the enzymes terminating the splitting from acetylcholine into acetic acid and choline and thus maintaining colinergic neurotransmission and improve cognition of Alzheimer’s patients (Jabir et al., 2018). The enzyme-inhibiting potential is revealed as colorless zones on a purple background. Acerola fruit (no. 1) showed a remarkably strong inhibition zone at *hR*_F_ 41 (Figures 2E,F). While roots and branches from the acerola tree are known to have anti-cholinesterase activity through the norfriedelins A-C (Liu et al., 2013), only cytotoxic, anti-HIV, antioxidant, antihyperglycemic, skin whitening, and antimicrobial activities are described for extracts from the fruits (Motohashi et al., 2004; Belwal et al., 2018a). This zone showed not only a strong response in the AChE and BChE assays, but also in most other assays (Figure 2). Other inhibitory zones were detected in ginger (no. 25, *hR*_F_ 99), kola (no. 31, *hR*_F_ 68), marjoram (no. 35, *hR*_F_ 99), yerba mate green (no. 37, *hR*_F_ 88), lemon balm (no. 38, *hR*_F_ 32), peppermint and rooibos (nos. 44 and 45, both *hR*_F_ 34), Siberian ginseng (no. 56, *hR*_F_ 93), and hawthorn (nos. 62–64, *hR*_F_ 60 and 85) (Figures 2–4E,F). Some typical traditional medicines used in the treatment of Alzheimer´s disease, such as *Panax notoginseng*, *Ginkgo biloba* (no. 14), *Melissa officinalis* (no. 38), and *Salvia officinalis* (no. 47), are also screened here. Among these, *Melissa officinalis* (no. 38) possess cholinesterase-inhibiting potential, the others operate according to a different mechanism to treat the neurodegenerative disease (Sharma et al., 2019).

#### 3.2.3 Compounds Inhibiting β-Glucuronidase

The detoxification mechanism via glucuronidation can be reversed with β-glucuronidase from opportunistic *Enterobacteriaceae* such as *Escherichia coli*, resulting in gastrointestinal malfunction. This can be prevented by inhibiting the microbial β-glucuronidase, where such inhibitors address the extra loop in the bacterial enzyme (in contrast to the mammalian one) (Mahran et al., 2020). The enzyme inhibitors do not cleave the chromogenic substrate and are therefore detected as colorless zones on an indigo-blue background. In each eucalyptus (no. 9), guarana (no. 16), marjoram (no. 35), yerba mate green (no. 37), oregano (no. 42), Siberian ginseng (no. 56), and cinnamon bark (no. 66), the whole sample track appeared white on the indigo-blue background due to the comparatively high abundance of β-glucuronidase inhibitors (Figures 2–4G). If the application volume is reduced by a factor of 4 for these botanicals, the individual inhibitors become evident (Supplementary Figure S2). All botanical extracts showed β-glucuronidase inhibitory potential at least in the solvent front (*hR*_F_ 99). For such screening results, repetition using a mobile phase of reduced solvent strength is recommended in order to better differentiate the individual inhibitors (Supplementary Figure S3C,D). Some isolated flavonoid standards, *i.e.,* isorhamnetin, kaempferol, liquiritigenin, daidzein, *etc.,* already proved to be active against β-glucuronidase (Sun et al., 2020). Their activity and also that of additional flavonoids have been confirmed by our study directly in herbs and spices.

#### 3.2.4 Compounds Inhibiting Tyrosinase

Polyphenoloxidases are responsible for the browning of cut or injured fruits or plant tissues. In mammalian cells, the corresponding tyrosinase controls melanogenesis by catalyzing the hydroxylation of phenols with subsequent oxidation to quinones. An overproduction of melanin induces pigmentary abnormality, freckles, or age spots. Preferably, the cosmetics industry is interested in naturally derived tyrosinase inhibitors (Taibon et al., 2015). In this context, ethnobotanicals are brought into focus. In South Africa, herbal extracts are traditionally used as skin care products to treat burns, abcesses, wounds and acne (Lall and Kishore, 2014). Chinese herbal medicines with anti-tyrosinase activity are traditionally used as folk skin whiteners. Among the studied botanicals, *Ginkgo biloba* (no. 14), *Panax ginseng* (no. 15), and *Zingiber officinale* (no. 25) were reported to inhibit mushroom tyrosinase (Ye et al., 2010; Hu et al., 2020). Our screening results showed that ginseng (no. 15) and ginger (no. 25) played a minor role in tyrosinase inhibition compared to other botanicals. However, differences in effects can be caused for example by plant subspecies, climate, soil, environmental and agricultural conditions as well as extraction mode. In the planar assay, tyrosinase inhibitors are shown as colorless zones on a greyish-brown background. Many botanical extracts showed multiple tyrosinase inhibitors (Figures 2–4H). While acerola (no. 1) (Belwal et al., 2018a), ginkgo (no. 14) (Shu et al., 2020), licorice (no. 55) (Li et al., 2017), and hawthorn (nos. 62–64) (Rocchetti et al., 2020) are known for their anti-tyrosinase activity, the screening results proved similarly potent tyrosinase inhibitors in artichoke (no. 4), plantain (no. 35), yerba mate green (no. 37), rosemary (no. 46), and yarrow (no. 50).

#### 3.2.5 Compounds Inhibiting α- and β-Glucosidase

The enzymes α- and β-glucosidase hydrolyze the saccharide dimers and oligomers, as well as glucosides dependent on the anomeric glycosidic bond, into resorbable monomers such as glucose and into aglycones. In the treatment of hyperglycemic blood levels of type 2 diabetes patients, enzyme inhibitors are of therapeutic interest by reducing postprandial glucose uptake (Simões-Pires et al., 2009; Turkiewicz et al., 2019). Screening results showed several α- and β-glucosidase inhibitors as a specific pattern (at *hR*_F_ 42, 48, 62, and 80) for artichoke (no. 4) in both assays (Figures 2I,J). Although the α- (Turkiewicz et al., 2019) and β-glucosidase (Morlock et al., 2021a) inhibitory potential and chemical composition of extracts of different artichoke cultivars have already been described, the distinct bioactive components have been scarcely assigned in literature. In both glucosidase inhibition assays, samples of blackberry leaves (no. 8), yellow fruit tea (no. 11), red fruit tea (no. 12), elderflower (no. 22), yerba mate green (no. 37), horsetail (no. 49), plantain (no. 53), Siberian ginseng (no. 56), and lemon verbena (no. 68) showed a positive response at *hR*_F_ 42 (Figures 2–4I,J). In all of them, the same bioactive compound was assumed. Another remarkably similar active compound was observed at *hR*_F_ 94 for basil (no. 5), ginkgo (no. 14), European blueberry (no. 18), elderberry (no. 21), hop (no. 24), yerba mate green (no. 37), lemon balm (no. 38), oregano (no. 42), peppermint (no. 44), rosemary (no. 46), star anise (no. 54), Siberian ginseng (no. 56), thyme (no. 57), and cinnamon bark (no. 66). All of them showed both α- and β-glucosidase inhibitory activities. Worldwide more than 1,000 herbal remedies were traditionally deployed for the maintenance and treatment of high blood glucose levels and thus diabetes. Both, the ethnobotanicals used and their mode of application (as tincture or extract, orally or as infusion) differ between local communities (Cock et al., 2021). Since western medical treatment methods for type 2 diabetes focus on hypoglycemic drugs, such as insulin, ethnopharmacological remedies are considered to be safe and to have less toxic side effects. According to the theory of traditional Chinese medicine, flavonoids (Bai et al., 2019) and polyphenols (Umeno et al., 2016) are attributed to have antidiabetic effects via several mechanisms. Glycyrrhizic acid from *Glycyrrhiza glabra* (no. 55), apigenin and its derivates as well as quercetin, found in many botanicals (Table 2), are known to target α-glucosidase (Bai et al., 2019).

**TABLE 2:** NP-HPTLC-EDA-RP-HPLC-DAD-HESI-MS signals of bioactive zones and respective activity (X) in *B. subtilis* **(A)**, *A. fischeri* **(B)**, AChE/BChE **(C)**, α-/β-glucosidase **(D)**, β-glucuronidase **(E)**, tyrosinase **(F)**, pYAS **(G)**, pYES **(H)**, pYAAS **(I)**, pYAES **(J)** and α-amylase **(K)**. In blue: analytes and respective bioactivity, which were confirmed by a standard; in rose: rebutted ones.

No	Botanical	*hR*_F_ (±1)	RT [min]	UV λ_max_ [nm]	*m/z*	Mass signal	Tentative assignment	Literature	A	B	C	D	E	F	G	H	I	J	K
2	Horehound, white	90	7.38	—	431	[M−H]^−^	apigenin-*O*-glucoside	Amessis-Ouchemoukh et al. (2014)	X	X	X	X	X	X					X
					467	[M + Cl]^−^													
					455	[M + Na]^+^													
					471	[M + K]^+^													
4	Artichoke	42	3.27/3.91/4.43	234, 322	353	[M−H]^−^	**chlorogenic acid^b^**	Rejeb et al. (2020), Morlock et al. (2021a)			**X**	**X**		**X**					
					355	[M + H]^+^													
					372	[M + NH_4_]^+^													
					377	[M + Na]^+^													
					393	[M + K]^+^													
			6.30	—	461	[M + Cl]^−^	cynarascoloside C	Farag et al. (2013)			X	X	X	X					
					471	[M + HCOO]^−^													
					485	[M + H_3_C-COO]^−^													
					444	[M + NH_4_]^+^													
					449	[M + Na]^+^													
					465	[M + K]^+^													
			6.71	232, 276	519	[M−H]^−^	unknown caffeic acid conjugate	Schütz et al. (2004); El Senousy et al. (2014)			X	X	X	X					
					555	[M + Cl]^−^													
					538	[M + NH_4_]^+^													
					543	[M + Na]^+^													
					559	[M + K]^+^													
		58	6.79	240	447	[M−H]^−^	luteolin-7-*O*-glucoside (cynaroside)	Farag et al. (2013)		X	X	X	X						
					483	[M + Cl]^−^													
		82	6.84	238, 280	433	[M−H]^−^	naringenin-7-*O*-glucoside	Schütz et al. (2004)			X		X	X					
					469	[M + Cl]^−^													
					496	[M + NO_3_]^−^													
9	*Eucalyptus*	57	6.71	222, 261	497	[M−H]^−^	eucaglobulin	Boulekbache-Makhlouf et al. (2010)	X	X	X		X	X					
					521	[M + Na]^+^													
					537	[M + K]^+^													
			6.76	251, 352	447	[M−H]^−^	methyl ellagic acid pentose	Boulekbache-Makhlouf et al. (2010); Santos et al. (2011)	X	X	X		X	X					
					449	[M + H]^+^													
					466	[M + NH_4_]^+^													
					471	[M + Na]^+^													
					487	[M + K]^+^													
		78	7.54	240	519	[M−H]^−^	cypellocarpin C	Boulekbache-Makhlouf et al. (2010)			X		X						
		90	5.23/6.18	—	185	[M + H]^+^	methyl gallate	Santos et al. (2011)	X	X	X	X	X	X					
					202	[M + NH_4_]^+^													
					207	[M + Na]^+^													
					223	[M + K]^+^													
12	Fruit tea, red	41	3.74	—	353	[M−H]^−^	**chlorogenic acid^b^**				**X**	**X**		**X**					
		46	3.22/3.91/4.58	233, 326	353	[M−H]^−^	**chlorogenic acid^b^**				**X**	**X**		**X**					
					355	[M + H]^+^													
					372	[M + NH_4_]^+^													
					377	[M + Na]^+^													
					393	[M + K]^+^													
13	Galangal	99	5.99	237, 284	301	[M−H]^−^	**quercetin**	Zhou et al. (2018)	X	X	X	X	X	X		X	X		
					320	[M + NH_4_]^+^													
					325	[M + Na]^+^													
					341	[M + K]^+^													
			7.40	—	343	[M−H]^−^	5-hydroxy-1-(4-hydroxyphenyl)-7-(4-hydroxy-3-methoxyphenyl)-3-heptanone	Zhou et al. (2018)	X	X	X	X	X	X		X	X		
					379	[M + Cl]^−^													
					389	[M + HCOO]^−^													
					403	[M + H_3_C-COO]^−^													
					345	[M + H]^+^													
					362	[M + NH_4_]^+^													
					367	[M + Na]^+^													
					383	[M + K]^+^													
			7.54	236, 289	271	[M−H]^−^	**pinobanksin**	Zhou et al. (2018)			**X**	**X**		**X**					
			7.59	—	359	[M−H]^−^	5-hydroxy-1-(3,4-dihydroxyphenyl)-7-(4-hydroxy-3-methoxyphenyl)-3-heptanone	Zhou et al. (2018)	X	X	X	X	X	X		X	X		
					395	[M + Cl]^−^													
					405	[M + HCOO]^−^													
					419	[M + H_3_C-COO]^−^													
					361	[M + H]^+^													
					378	[M + NH_4_]^+^													
					383	[M + Na]^+^													
					399	[M + K]^+^													
			7.94	221, 280	327	[M−H]^−^	(4 E)-1,5-bis(4-hydroxyphenyl)-1-methoxy-2-(methoxymethyl)-4-pentene	Zhou et al. (2018)	X	X	X	X	X	X		X	X		
					363	[M + Cl]^−^													
					329	[M + H]^+^													
					346	[M + NH_4_]^+^													
					351	[M + Na]^+^													
					367	[M + K]^+^													
			8.14	237, 289	255	[M−H]^−^	pinocembrin	Zhou et al. (2018)	X	X	X	X	X	X		X	X		
					257	[M + H]^+^													
					279	[M + Na]^+^													
			8.37	236	283	[M−H]^−^	galangin-3-methyl ether	Zhou et al. (2018)	X	X	X	X	X	X		X	X		
					285	[M + H]^+^													
			8.37	235	269	[M−H]^−^	**galangin**	Krüger et al. (2017); Zhou et al. (2018)	X	X	X	X	**X**	**X**		X	X		
					271	[M + H]^+^													
			8.52	—	283	[M + H]^+^	dihydroyashabushiketol	Zhou et al. (2018)	X	X	X	X	X	X		X	X		
					300	[M + NH_4_]^+^													
					305	[M + Na]^+^													
					321	[M + K]^+^													
14	Ginkgo	46	4.03	230, 267	344	[M + NH_4_]^+^	bilobalide	Mauri et al. (1999), Niu et al. (2017)					X	X					X
					349	[M + Na]^+^													
					365	[M + K]^+^													
		93	6.42	—	439	[M−H]^−^	ginkgolide C	Chen et al. (2005); Ding et al. (2006); Niu et al. (2017)	X	X		X	X	X			X		
					475	[M + Cl]^−^													
					458	[M + NH_4_]^+^													
					463	[M + Na]^+^													
					479	[M + K]^+^													
			6.86	—	407	[M−H]^−^	**ginkgolide A**	Wang et al. (2016), Niu et al. (2017)	X	X		**X**	X	X			X		
					443	[M + Cl]^−^													
					453	[M + HCOO]^−^													
					467	[M + H_3_C-COO]^−^													
					815	[2M−H]^−^													
					426	[M + NH_4_]^+^													
					431	[M + Na]^+^													
					447	[M + K]^+^													
			6.86	—	423	[M−H]^−^	**ginkgolide B**	Wang et al. (2016), Niu et al. (2017)	X	X		**X**	X	X			X		
					459	[M + Cl]^−^													
					847	[2M−H]^−^													
					442	[M + NH_4_]^+^													
					463	[M + K]^+^													
15	Ginseng	16	7.60	243	836	[M + Cl]^−^	ginsenoside Rg1/Rf	Du et al. (2018); Wilson and Sander (2018)					X				X		X
					846	[M + HCOO]^−^													
					860	[M + H_3_C-COO]^−^													
					824	[M + Na]^+^													
					840	[M + K]^+^													
		24	8.08	—	800	[M−H]^−^	ginsenoside Rg1/Rf	Du et al. (2018), Wilson and Sander (2018)					X				X		
					836	[M + Cl]^−^													
					846	[M + HCOO]^−^													
					860	[M + H_3_C-COO]^−^													
					824	[M + Na]^+^													
					840	[M + K]^+^													
16	Guarana	91	5.12/5.98	203, 279	289	[M−H]^−^	**(epi)catechin**	da Silva et al. (2017), Morlock et al. (2021b)		X			X	**X**			X	**X**	
					325	[M + Cl]^−^													
					291	[M + H]^+^													
					329	[M + K]^+^													
			4.23	205, 281	577	[M−H]^−^	B-type procyanidin dimer	da Silva et al. (2017)		X			X	X			X	X	
					579	[M + H]^+^													
					601	[M + Na]^+^													
					617	[M + K]^+^													
19	*Hibiscus*	45	4.57	234, 322	353	[M−H]^−^	**chlorogenic acid^b^**				**X**	**X**	X	**X**					
22	Elder flower	40	3.80/4.67	233, 326	353	[M−H]^−^	**chlorogenic acid^b^**				**X**	**X**		**X**					
					355	[M + H]^+^													
					372	[M + NH_4_]^+^													
					377	[M + Na]^+^													
					393	[M + K]^+^													
24	Hop	92	7.05	264, 347	447	[M−H]^−^	kaempferol-3-*O*-glucoside (astragalin)	Önder et al. (2013)	X	X			X						
					449	[M + H]^+^													
					471	[M + Na]^+^													
					487	[M + K]^+^													
		94	7.83	254, 324	317	[M−H]^−^	cohulupone	Önder et al. (2013), Sommella et al. (2018)	X	X	X	X	X	X		X			
					319	[M + H]^+^													
					336	[M + NH_4_]^+^													
					341	[M + Na]^+^													
					357	[M + K]^+^													
25	Ginger	94	8.06	297	293	[M−H]^−^	6-gingerol	Krüger et al. (2018)	X	X	X	X	X			X			
					295	[M + H]^+^													
					312	[M + NH_4_]^+^													
					317	[M + Na]^+^													
					333	[M + K]^+^													
28	Chamomile	50	6.95	237, 266, 335	431	[M−H]^−^	apigenin-7-*O*-glucoside	Lin and Harnly, (2012)					X	X					
					433	[M + H]^+^													
					455	[M + Na]^+^													
					471	[M + K]^+^													
		63	7.51	241, 257, 331	473	[M−H]^−^	apigenin-7-*O*-(2″-*O*-acetylglucoside)	Lin and Harnly, (2012)					X			X			
					475	[M + H]^+^													
					497	[M + Na]^+^													
					513	[M + K]^+^													
33	Caraway	43	7.04	—	447	[M−H]^−^	luteolin-7-*O* glucoside						X	X					
					471	[M + Na]^+^													
					487	[M + K]^+^													
35	Marjoram	78	6.52	236, 278	433	[M−H]^−^	quercetin arabinoside	Hossain et al. (2014)		X			X	X		X			
					469	[M + Cl]^−^													
					452	[M + NH_4_]^+^													
					457	[M + Na]^+^													
					473	[M + K]^+^													
		93	6.20	219, 326	359	[M−H]^−^	**rosmarinic acid**	Hossain et al. (2014), Çelik et al. (2017)		X	**X**	X	X	X					
					395	[M + Cl]^−^													
					719	[2M−H]^−^													
					378	[M + NH_4_]^+^													
					383	[M + Na]^+^													
					399	[M + K]^+^													
		97	7.85	235, 287	301	[M−H]^−^	hesperetin/**quercetin**	Hossain et al. (2014); Erenler et al. (2016)	X	X	**X**	X	X	**X**					
					337	[M + Cl]^−^													
					303	[M + H]^+^													
37	Yerba mate green	6	3.90	—	343	[M + H]^+^	dicaffeic acid	Souza et al. (2011)	X	X	X	X	X	X					
					365	[M + Na]^+^													
					381	[M + K]^+^													
			3.90	—	452	[M + NH_4_]^+^	quercetin arabinoside	Souza et al. (2011)	X	X	X	X	X	X					
					457	[M + Na]^+^													
					473	[M + K]^+^													
		14	7.07	—	482	[M + NH_4_]^+^	quercetin glucoside	Bravo et al. (2007); Souza et al. (2011), Mateos et al. (2018)	X	X	X	X	X	X					
					487	[M + Na]^+^													
					503	[M + K]^+^													
			4.94/5.32	236, 324	341	[M−H]^−^	dicaffeic acid	Bravo et al. (2007); Souza et al. (2011)	X	X	X	X	X	X					
					364	[M + Na]^+^													
					381	[M + K]^+^													
		20	6.84	256, 356	609	[M−H]^−^	**rutin**	Krüger et al. (2017), Mateos et al. (2018)	X	X	**X**	X	X	**X**					
					611	[M + H]^+^													
					633	[M + Na]^+^													
					649	[M + K]^+^													
		40	2.75/3.65/4.42	217, 324	353	[M−H]^−^	**chlorogenic acid^b^**	Mateos et al. (2018), Morlock et al. (2021a)	X	X	**X**	**X**	X	**X**					
					355	[M + H]^+^													
					372	[M + NH_4_]^+^													
					377	[M + Na]^+^													
					393	[M + K]^+^													
		47	5.11	236, 322	367	[M−H]^−^	feruloylquinic acid	Bravo et al., 2007, Souza et al. (2011), Mateos et al. (2018)		X		X	X	X					
					369	[M + H]^+^													
					391	[M + Na]^+^													
					407	[M + K]^+^													
		85	6.20/6.39/6.48	220, 240, 327	515	[M−H]^−^	dicaffeoylquinic acid	Krüger et al. (2017), Mateos et al. (2018)			X	X	X	X					
					517	[M + H]^+^													
					539	[M + Na]^+^													
					555	[M + K]^+^													
38	Lemon balm	92	6.21	219, 326	359	[M−H]^−^	**rosmarinic acid**	Krüger et al. (2017), Yilmaz (2020)		X	**X**	X	X	X					
					395	[M + Cl]^−^													
					719	[2M−H]^−^													
					361	[M + H]^+^													
					378	[M + NH_4_]^+^													
					383	[M + Na]^+^													
					399	[M + K]^+^													
41	Orange peel	21	5.74	229, 278	390	[M + NH_4_]^+^	sinensetin/tangeretin	Anagnostopoulou et al. (2005); Li et al. (2006)		X	X	X	X	X					
					395	[M + Na]^+^													
					411	[M + K]^+^													
			6.83	231,283	593	[M−H]^−^	didymin	Anagnostopoulou et al. (2005)		X	X	X	X	X					
					629	[M + Cl]^−^													
					595	[M + H]^+^													
					617	[M + Na]^+^													
					633	[M + K]^+^													
			7.06	227, 268, 339	577	[M−H]^−^	apigenin-7-*O*-rutinoside (isorhoifolin)	Anagnostopoulou et al. (2005)		X	X	X	X	X					
					613	[M + Cl]^−^													
					579	[M + H]^+^													
					601	[M + Na]^+^													
					617	[M + K]^+^													
			7.06	227, 268, 339	607	[M−H]^−^	diosmin	Anagnostopoulou et al. (2005)		X	X	X	X	X					
					643	[M + Cl]^−^													
					609	[M + H]^+^													
					631	[M + Na]^+^													
					647	[M + K]^+^													
		34	6.63	232, 280	595	[M−H]^−^	**eriocitrin**	Manthey and Grohmann (1996), Anagnostopoulou et al. (2005)			**X**			X			X	**X**	
					631	[M + Cl]^−^													
					658	[M + NO_3_]^−^													
					597	[M + H]^+^													
					619	[M + Na]^+^													
					635	[M + K]^+^													
			6.87	220, 284	579	[M−H]^−^	**naringin**	Anagnostopoulou et al. (2005); Sawalha et al. (2009), Puranik et al. (2019)			X			X			X	**X**	
					615	[M + Cl]^−^													
					642	[M + NO_3_]^−^													
					581	[M + H]^+^													
					603	[M + Na]^+^													
					619	[M + K]^+^													
			6.98	284	609	[M−H]^−^	**hesperidin**	Anagnostopoulou et al. (2005), Sawalha et al. (2009), Puranik et al. (2019)			X			X			X	**X**	
					645	[M + Cl]^−^													
					672	[M + NO_3_]^−^													
					611	[M + H]^+^													
					633	[M + Na]^+^													
					649	[M + K]^+^													
		^a^	7.05	—	261	[M + H]^+^	**meranzin**	Dugo et al. (2000)			X			X			X	X	
					278	[M + NH_4_]^+^													
		92	8.23	249, 269, 335	403	[M + H]^+^	hexamethoxyflavone (nobiletin)	Anagnostopoulou et al. (2005), Li et al. (2006)			X		X		X			X	
					425	[M + Na]^+^													
					441	[M + K]^+^													
					827	[2M + Na]^+^													
			8.44	—	373	[M + H]^+^	sinensetin/tangeretin/	Anagnostopoulou et al. (2005), Li et al. (2006)			X		X		X			X	
					395	[M + Na]^+^													
					411	[M + K]^+^													
42	Oregano	32^a^	2.88	219, 270	304	[M + NH_4_]^+^	luteolin/**kaempferol**	Hossain et al. (2010); Vallverdú-Queralt et al. (2014)		X			X	X					
					309	[M + Na]^+^													
					325	[M + K]^+^													
		62	7.38	241	331	[M−H]^−^	carnosic acid	Hossain et al. (2010)		X	X	X	X						
					367	[M + Cl]^−^													
					377	[M + HCOO]^−^													
					391	[M + H_3_C-COO]^−^													
					333	[M + H]^+^													
					350	[M + NH_4_]^+^													
					355	[M + Na]^+^													
					371	[M + K]^+^													
		81	5.81/6.20	—	379	[M + Cl]^−^	rosmadial	Hossain et al. (2010)	X		X								
					367	[M + Na]^+^													
					383	[M + K]^+^													
		93	5.99	238	331	[M + H]^+^	carnosol	Hossain et al. (2010)	X	X	X	X	X	X					
					353	[M + Na]^+^													
					369	[M + K]^+^													
			7.97	221, 268	331	[M−H]^−^	carnosic acid	Hossain et al. (2010)	X	X	X	X	X	X					
					367	[M + Cl]^−^													
					333	[M + H]^+^													
					371	[M + K]^+^													
		99	7.51	238, 279	271	[M−H]^−^	**naringenin**	Krüger et al. (2017), Mbachu et al. (2020)	X	X	**X**		X			X			
			7.79	239	269	[M−H]^−^	**apigenin**	Hossain et al. (2010), Mbachu et al. (2020)	X	X	**X**		**X**			X			
					271	[M + H]^+^													
			7.79	239	315	[M−H]^−^	3-*O*-methylquercetin **(isorhamnetin)**	Hossain et al. (2010)	X	X	**X**		X			X			
					317	[M + H]^+^													
					339	[M + Na]^+^													
					355	[M + K]^+^													
			8.01	221, 268	313	[M−H]^−^	cirsimaritin	Hossain et al. (2010), Krüger et al. (2017)	X	X	X		X			X			
					315	[M + H]^+^													
					337	[M + Na]^+^													
					353	[M + K]^+^													
44	Peppermint	20	7.05	249, 344	607	[M−H]^−^	diosmin	Fecka et al. (2004)		X	X			X					
					609	[M + H]^+^													
					631	[M + Na]^+^													
					647	[M + K]^+^													
		26	7.00	267, 267, 339	577	[M−H]^−^	apigenin-7-*O*-rutinoside (isorhoifolin)	Hawrył, (2014)		X	X	X	X						
					613	[M + Cl]^−^													
					579	[M + H]^+^													
					601	[M + Na]^+^													
					617	[M + K]^+^													
		93	6.23	235, 286	359	[M−H]^−^	**rosmarinic acid**	Fecka et al. (2004)	X	X	X	X	X	X					
					395	[M + Cl]^−^													
					383	[M + Na]^+^													
					399	[M + K]^+^													
			6.99	236	577	[M−H]^−^	apigenin-7-*O*-rutinoside (isorhoifolin)	Hossain et al. (2010)	X	X	**X**	X	X	X					
					613	[M + Cl]^−^													
					640	[M + NO_3_]^−^													
					579	[M + H]^+^													
					601	[M + Na]^+^													
					617	[M + K]^+^													
46	Rosemary	25	4.89	232, 269	337	[M + Na]^+^	cirsimaritin	Ezzat et al. (2016), Krüger et al. (2017), Pérez-Mendoza et al. (2020)		X	X	X	X	X					
					353	[M + K]^+^													
			5.44	226, 285	362	[M + NH_4_]^+^	rosmadial	Mena et al. (2016), Pérez-Mendoza et al. (2020)		X	X	X	X	X					
					367	[M + Na]^+^													
					383	[M + K]^+^													
			5.78	224, 263	387	[M−H]^−^	medioresinol	Mena et al. (2016)		X	X	X	X	X					
					406	[M + NH_4_]^+^													
					411	[M + Na]^+^													
					427	[M + K]^+^													
		^a^	6.06	233	315	[M−H]^−^	3-*O*-methylquercetin **(isorhamnetin)**	Mena et al. (2016)		X	X	X	X	X					
					351	[M + Cl]^−^													
					361	[M + HCOO]^−^													
					375	[M + H_3_C-COO]^−^													
					317	[M + H]^+^													
					339	[M + Na]^+^													
					355	[M + K]^+^													
			6.13	235	499	[M + Cl]^−^	quercetin-3-*O*-hexoside (isoquercitrin)	Hossain et al. (2010)		X	X	X	X	X					
					503	[M + K]^+^													
			6.99	236	577	[M−H]^−^	apigenin-7-*O*-rutinoside (isorhoifolin)	Mena et al. (2016)		X	X	X	X	X					
					613	[M + Cl]^−^													
					579	[M + H]^+^													
					601	[M + Na]^+^													
					617	[M + K]^+^													
		41	4.05/5.05	220, 263	299	[M−H]^−^	diosmetin/6-*O*-methylapigenin (hispidulin)	Pérez-Mendoza et al. (2020)			X	X	X						
					335	[M + Cl]^−^													
					301	[M + H]^+^													
					318	[M + NH_4_]^+^													
					323	[M + Na]^+^													
					339	[M + K]^+^													
		62^a^	6.48	—	319	[M + Cl]^−^	7-*O*-methylapigenin (genkwanin)/**acacetin**	Ezzat et al. (2016), Mena et al. (2016), Krüger et al. (2017), Pérez-Mendoza et al. (2020)		X	X	X	X	X					
					302	[M + NH_4_]^+^													
					307	[M + Na]^+^													
					323	[M + K]^+^													
			6.89	236	461	[M−H]^−^	luteolin-7-*O*-glucuronide	Mena et al. (2016), Pérez-Mendoza et al. (2020)		X	X	X	X	X					
					463	[M + H]^+^													
					485	[M + Na]^+^													
					501	[M + K]^+^													
			7.23	—	377	[M + Cl]^−^	caffeic acid hexoside	Hossain et al. (2010)		X	X	X	X	X					
					401	[M + H_3_C-COO]^−^													
					365	[M + Na]^+^													
					381	[M + K]^+^													
		92	6.19	326	359	[M−H]^−^	**rosmarinic acid**	Hossain et al. (2010), Mena et al. (2016), Pérez-Mendoza et al. (2020)	X	X	**X**	X	X	X					
					395	[M + Cl]^−^													
					719	[2M−H]^−^													
					378	[M + NH_4_]^+^													
					383	[M + Na]^+^													
					399	[M + K]^+^													
					743	[2M + Na]^+^													
			8.06	—	331	[M−H]^−^	carnosic acid	Ezzat et al. (2016), Mena et al. (2016), Pérez-Mendoza et al. (2020)	X	X	X	X	X	X					
					333	[M + H]^+^													
					355	[M + Na]^+^													
					371	[M + K]^+^													
		99	6.79	—	287	[M + H]^+^	luteolin	Mena et al. (2016)	X	X	X	X	X	X					
					309	[M + Na]^+^													
					325	[M + K]^+^													
48	Sea buckthorn	21	6.74/7.02	254, 354	623	[M−H]^−^	isorhamnetin-3-*O*-rutoside (narcissin)/isorhamnetin-3-glucoside-7-rhamnoside (brassidin)	Zheng et al. (2016)					X	X					
					659	[M + Cl]^−^													
					669	[M + HCOO]^−^													
					625	[M + H]^+^													
					647	[M + Na]^+^													
					663	[M + K]^+^													
50	Yarrow	40	3.80	221, 325	353	[M−H]^−^	**chlorogenic acid^b^**	Giorgi et al. (2009)	X	X	**X**	**X**	X	**X**					
					355	[M + H]^+^													
					377	[M + Na]^+^													
					393	[M + K]^+^													
55	Licorice	31	6.52/6.63	217, 228, 271	549	[M−H]^−^	**liquiritin apioside**	Wong et al. (2018)	X	X	**X**	X	X	X			X	**X**	
					585	[M + Cl]^−^													
			6.52/6.63	218, 276	257	[M + H]^+^	2′,4′,4′-trihydroxychalcone	Li et al. (2016)	X				X	X			X	X	
			7.10	250	561	[M−H]^−^	glycyroside	Montero et al. (2016); Wong et al. (2018)	X				X	X			X	X	
					597	[M + Cl]^−^													
					621	[M + H_3_C-COO]^−^													
					563	[M + H]^+^													
					585	[M + Na]^+^													
					601	[M + K]^+^													
			7.85	251	821	[M−H]^−^	**glycyrrhizic acid**	Kong et al. (2014); Li et al. (2016), Krüger et al. (2017)				X		X			X	**X**	
					843	[M−2H + Na]^−^													
					859	[M−2H + K]^−^													
		49	6.64	—	549	[M−H]^−^	**liquiritin apioside**	Krüger et al. (2017), Wong et al. (2018)	X	X	**X**	X	X	X			X	**X**	
					257	[M−C_5_H_8_O_4_+H]^+^													
					419	[M−C_11_H_18_O_9_+H]^+^													
					573	[M + Na]^+^													
					589	[M + K]^+^													
		62	7.15	249	695	[M−H]^−^	licorice glycoside B/D1/D2	Wong et al. (2018)						X					X
					719	[M + Na]^+^													
					735	[M + K]^+^													
		77	6.49/7.15/7.24	234, 268, 372	417	[M−H]^−^	(iso)liquiritin	Krüger et al. (2017), Wong et al. (2018)		X	X			X	X	X			X
					453	[M + Cl]^−^													
					419	[M + H]^+^													
					441	[M + Na]^+^													
					457	[M + K]^+^													
		93	7.17	218, 276	255	[M−H]^−^	**(iso)liquiritigenin**	Kong et al. (2014), Boonmuen et al. (2016), Li et al. (2016), Montero et al. (2016)	X	X	X	X	X	X		**X**			
					257	[M + H]^+^													
					279	[M + Na]^+^													
			7.41	—	253	[M−H]^−^	**daidzein**	Liu et al. (2001), Nomura et al. (2002), Cornwell et al. (2004)	X	X	X	X	X	X		**X**			
					255	[M + H]^+^													
					277	[M + Na]^+^													
					293	[M + K]^+^													
			7.41	—	283	[M−H]^−^	biochanin A	Liu et al. (2001)	X	X	X	X	X	X					
					285	[M + H]^+^													
					307	[M + Na]^+^													
					323	[M + K]^+^													
			7.91	—	267	[M−H]^−^	coumestrol	Cornwell et al. (2004)	X	X	X	X	X	X		X			
					269	[M + H]^+^													
					291	[M + Na]^+^													
					307	[M + K]^+^													
56	Siberian ginseng	14	6.48	—	499	[M + Cl]^−^	quercetin-3-*O*-galactoside (hyperoside)/quercetin-3-*O*-glucopyranoside (isoquercitrin)	Wang et al. (2019)	X	X		X	X	X		X			
					423	[M + H_3_C-COO]^−^													
					487	[M + Na]^+^													
					503	[M + K]^+^													
		36	2.81/3.68/4.45/4.66	217, 324	353	[M−H]^−^	**chlorogenic acid^b^**	Wang et al. (2019)	X	X	**X**	**X**	X	**X**					
					355	[M + H]^+^													
					372	[M + NH_4_]^+^													
					377	[M + Na]^+^													
					393	[M + K]^+^													
			5.40	—	439	[M−H]^−^	akebonoic acid	Ge et al. (2017)	X	X	**X**	**X**	X	**X**					
					475	[M + Cl]^−^													
					499	[M + H_3_C-COO]^−^													
					463	[M + Na]^+^													
					479	[M + K]^+^													
			7.38	—	469	[M + Cl]^−^	naringenin-7-*O*-glucoside (prunin)	Kuźniewski et al. (2018)	X	X	X	X	X	X					
					479	[M + HCOO]^−^													
					493	[M + H_3_C-COO]^−^													
					452	[M + NH_4_]^+^													
					457	[M + Na]^+^													
					473	[M + K]^+^													
			7.78	—	483	[M + Cl]^−^	kaempferol-3-*O*-glucoside (astragalin)/quercetin-3-*O*-rhamnoside (quercitrin)	Kuźniewski et al. (2018), Wang et al. (2019)	X	X	X	X	X	X					
					493	[M + HCOO]^−^													
					507	[M + H_3_C-COO]^−^													
					466	[M + NH_4_]^+^													
					471	[M + Na]^+^													
					487	[M + K]^+^													
57	Thyme	22	6.62	235	557	[M + Cl]^−^	rosmarinyl glucoside (rosmarinic acid-*O*-hexoside)	Vallverdú-Queralt et al. (2014)	X	X	X	X	X	X					
					540	[M + NH_4_]^+^													
					545	[M + Na]^+^													
					561	[M + K]^+^													
		31	4.36	—	355	[M + H]^+^	**chlorogenic acid^b^**	Hossain et al. (2010), Vallverdú-Queralt et al. (2014)	X	X	**X**	**X**	X	**X**					
			5.78	—	387	[M−H]^−^	medioresinol	Hossain et al. (2010)	X	X	X	X	X	X					
		61	7.36	237, 293	355	[M + Na]^+^	carnosic acid	Hossain et al. (2010), Vallverdú-Queralt et al. (2014)	X	X	X	X	X	X					
					371	[M + K]^+^													
		64^a^	6.46	—	302	[M + NH_4_]^+^	7-*O*-methylapigenin (genkwanin)/**acacetin**	Hossain et al. (2010)	X	X	X	X	X	X					
					307	[M + Na]^+^													
					323	[M + K]^+^													
		75	7.15	238	344	[M + NH_4_]^+^	coumaroyl hexoside (coumaric acid-*O*-hexoside)	Vallverdú-Queralt et al. (2014)	X	X	X	X	X	X					
					349	[M + Na]^+^													
					365	[M + K]^+^													
		91	6.13	326	359	[M−H]^−^	**rosmarinic acid**	Krüger et al. (2017)	X	X	**X**	X	X	X					
					395	[M + Cl]^−^													
					361	[M + H]^+^													
					378	[M + NH_4_]^+^													
					383	[M + Na]^+^													
					399	[M + K]^+^													
58	Grape seed	92	5.13	204, 279	289	[M−H]^−^	**(epi)catechin**	Yilmaz and Toledo (2004)	X	X		X	X	**X**			X	**X**	
					325	[M + Cl]^−^													
					291	[M + H]^+^													
					329	[M + K]^+^													
62–64	Hawthorn	41	6.14	234, 280	359	[M−H]^−^	lariciresinol/isolariciresinol/cyclolariciresinol	Huang et al. (2018), Rocchetti et al. (2020)	X	X	X		X	X					
					395	[M + Cl]^−^													
			6.54	—	421	[M + Cl]^−^	dimethylmatairesinol	Rocchetti et al. (2020)	X	X	X		X	X					
					409	[M + Na]^+^													
					425	[M + K]^+^													
			6.80/7.13	230, 268, 336	619	[M−H]^−^	4‴-acetylvitexin-2″-*O*-rhamnoside	Krüger et al. (2017)	X	X	X		X	X					
					655	[M + Cl]^−^													
					621	[M + H]^+^													
					643	[M + Na]^+^													
					659	[M + K]^+^													
		58	4.70/5.32	234, 310	337	[M−H]^−^	3-*p*-coumaroylquinic acid/4-*p*-coumaroylquinic acid/5-*p*-coumaroylquinic acid/	Rocchetti et al. (2020)			X		X	X					
					339	[M + H]^+^													
					356	[M + NH_4_]^+^													
					361	[M + Na]^+^													
					377	[M + K]^+^													
			6.82	255, 355	463	[M−H]^−^	isorhamnetin-7-*O*-rhamnoside	Rocchetti et al. (2020)			X		X	X					
					465	[M + H]^+^													
					487	[M + Na]^+^													
					503	[M + K]^+^													
			7.64	—	417	[M−H]^−^	syringaresinol/oleoside dimethylester	Rocchetti et al. (2020)			X		X	X					
					453	[M + Cl]^−^													
					463	[M + HCOO]^−^													
					477	[M + H_3_C-COO]^−^													
					441	[M + Na]^+^													
					457	[M + K]^+^													
		98	7.24	—	286	[M−H]^−^	cyanidin	Rocchetti et al. (2020)	X	X	X			X		X			
					288	[M + H]^+^													
					326	[M + K]^+^													
			7.60	—	197	[M−H]^−^	**syringic acid**	Rocchetti et al. (2020)	X	X	**X**			X		X			
					199	[M + H]^+^													
					221	[M + Na]^+^													
					237	[M + K]^+^													
			8.09	—	305	[M−H]^−^	(+)-gallocatechin/(−)-epigallocatechin	Rocchetti et al. (2020)	X	X	X			X		X			
					341	[M + Cl]^−^													
					307	[M + H]^+^													
					329	[M + Na]^+^													
					345	[M + K]^+^													
			8.65		517	[M−H]^−^	6″-*O*-malonylgenistin	Rocchetti et al. (2020)	X	X	X			X		X			
					541	[M + Na]^+^													
					557	[M + K]^+^													
			8.65	—	487	[M−H]^−^	luteolin-6-C-glucoside/6″-*O*-acetylglycitin	Rocchetti et al. (2020)	X	X	X			X		X			
					489	[M + H]^+^													
					506	[M + NH_4_]^+^													
					511	[M + Na]^+^													
					527	[M + K]^+^													
			9.27	-	471	[M−H]^−^	crataegolic acid (maslinic acid)	Vierling et al. (2003)	X	X	X			X		X			
					495	[M + Na]^+^													
					511	[M + K]^+^													
67	Lemon peel	95	7.46	—	469	[M−H]^−^	limonin	Baldi et al. (1995)		X			X	X	X	X			X
					505	[M + Cl]^−^													
					515	[M + HCOO]^−^													
					529	[M + H_3_C-COO]^−^													
					471	[M + H]^+^													
					488	[M + NH_4_]^+^													
					493	[M + Na]^+^													
					509	[M + K]^+^													
68	Lemon verbena	42	6.51	—	639	[M−H]^−^	β-OH-(iso)verbascoside	Bilia et al. (2008)	X	X	X	X	X	X				X	
					663	[M + Na]^+^													
					679	[M + K]^+^													
			6.66/6.82/6.93	222, 330	623	[M−H]^−^	(iso)verbascoside	Bilia et al. (2008), Quirantes-Piné et al. (2010), Krüger et al. (2017)	X	X	X	X	X	X				X	
					642	[M + NH_4_]^+^													
					647	[M + Na]^+^													
					663	[M + K]^+^													

a Standard eluted at another *hR*_F_, value.

b Or neochlorogenic or cryptochlorogenic acid.

#### 3.2.6 Compounds With Agonistic/Antagonistic Hormonal Effects

Disrupting endocrine signaling pathways can have a severe impact on hormonal balance and cause feminization/masculinization, infertility, acne, and menstrual cycle disorders. Also phytochemicals present in food (Morlock and Klingelhöfer, 2014) or commodities in daily use (Klingelhöfer et al., 2020) can affect the human hormone system. HPTLC hyphenated to the planar yeast androgen/estrogen screens (pYAS/pYES) and their antagonistic versions (pYAAS/pYAES) showed positive reactions to a limited extent (Figures 2–4K–N). In the pYAS bioassay, a few 4-methylumbelliferone-blue and thus androgenic responses were detected in acerola (no. 1, *hR*_F_ 16), elderberry (no. 21, *hR*_F_ 72), chamomile (no. 28, *hR*_F_ 99), orange peel (no. 41, *hR*_F_ 67 and 98), horsetail (no. 49, *hR*_F_ 49 and 61), lemon peel (no. 67, *hR*_F_ 99), and for several samples at the application zone (nos. 4, 15, 25, 30, and 56). In the pYES bioassay, 15 botanicals showed estrogen-like activity. In some samples, phytoestrogens were known and expected, *e.g.,* 8-prenylnaringenin in hop (no. 24) (Prencipe et al., 2014; Mbachu et al., 2020), (iso-)liquiritigenin in licorice (no. 55) (Boonmuen et al., 2016), or pesticide residues from fruit surfaces (Schulte-Oehlmann et al., 2011) such as orange peel (no. 41), grape peel (no. 59), or lemon peel (no. 67). However, in acerola (no. 1, *hR*_F_ 15 and 27), galangal (no. 13, *hR*_F_ 99), chamomile (no. 28, *hR*_F_ 63 and 95), lovage (no. 34, *hR*_F_ 97), marjoram (no. 35, *hR*_F_ 62), oregano (no. 42, *hR*_F_ 45), juniper (no. 60, *hR*_F_ 89), grape leaves (no. 61, *hR*_F_ 92), and hawthorn (nos. 62–64, *hR*_F_ 94), estrogen-like responses were also detected. Since steroid hormone-like compounds are known to have a greater affinity to hERα, but several phytoestrogens (*e.g.,* daidzein or genistein) to hERβ (Mbachu et al., 2020), the pYES bioassay was also performed via the hERβ for the first time. The assay was analogously run for both receptors, but no remarkable difference was observed in the results obtained by both (Figure 5A versus Figure 5B). The hERα seemed to be less selective. However, a significant difference in the estrogenic pattern was observed after metabolization with the S9 mixture (Figure 5C). The use of genetically modified yeast cells containing the hERβ for the pYES bioassay and the simulated metabolization via the S9 liver enzyme system were reported here for the first time.

In the respective antagonistic assays, only a few zones were detected. For acerola (no. 1, *hR*_F_ 99), fenugreek (no. 6, *hR*_F_ 99), eucalyptus (no. 9, *hR*_F_ 5 or 89), ginseng (no. 15, *hR*_F_ 96), guarana (no. 16, *hR*_F_ 60), kola (no. 31, *hR*_F_ 73), orange peel (no. 41, *hR*_F_ 25 or 32), licorice (no. 55, *hR*_F_ 27), thyme (no. 57, *hR*_F_ 24 or 50), and lemon verbena (no. 68, *hR*_F_ 99), the possibly antiandrogenic zones were also investigated using an overlayed area of the fluorescent 4-methylumbelliferone (Supplementary Figure S4) to exclude any false-positive response as observed for the physicochemical fluorescence reduction by pigments or dyes (Klingelhöfer et al., 2020). Seven botanical samples (nos. 6, 15, 16, 31, 41, 55, and 68) showed truly antiandrogenic activities. The same verification test was run for possible antiestrogens in galangal (no. 13, *hR*_F_ 99), guarana (no. 16, *hR*_F_ 72 or 91), garlic (no. 30, *hR*_F_ 99), kola (no. 31, *hR*_F_ 99), orange peel (no. 41, *hR*_F_ 34), licorice (no. 55, *hR*_F_ 31 or 43), thyme (no. 57, *hR*_F_ 31), grape seeds/leaves (nos. 58/61, *hR*_F_ 48, 61 or 92), and lemon verbena (no. 68, *hR*_F_ 99). Seven botanicals (nos. 16, 30, 41, 55, 57, 58, and 68) revealed true antiestrogenic properties (Supplementary Figure S5). In the antiestrogenic assay, synergistic effects were evident. The overlapping 17-β-estradiol area (10 pg/70 mm area) is partially enhanced on sample tracks, apparent as a cotton swab shape if compared to the solvent blank (Figures 2–4N). This observation revealed synergistic effects between distinct botanical ingredients in most samples (except for nos. 7, 29, and 31) and 17-β-estradiol, resulting in fortified estrogenic activities (Figure 6). This overlapped experimental setup can be transferred to all other assays to identify synergy. Such a synergistic effect detection strategy was reported here for the first time.

#### 3.2.7 Compounds With Genotoxic Effects

In recent decades, there has been a steady trend away from industrial medical care towards phytotherapy based on medicinal herbs, which have been used in traditional medicine for years. The common belief that phytochemicals are gentler than synthetic medicines may prove to be a fallacy, as most toxic compounds originate in nature (Efferth and Kaina, 2011). The SOS-Umu-C assay is a reporter gene assay indicating genotoxicity based on a genetically modified test organism *Salmonella typhimurium* TA1535 [pSK1002]. Under genotoxic stress, the SOS-DNA repair mechanism is induced. The SOS-Umu response activates the *lacZ* gene, encoding for β-galactosidase, which enables substrate cleavage into detectable products (Meyer et al., 2020). As the analyzed botanicals are commonly used as spices, in herbal medicine, or as tea, no genotoxic substances were expected. Nevertheless, pyrrolizidine alkaloids are known for genotoxic properties and their occurrence in herbal formulations (Habs et al., 2017), *e.g.,* only a few micrograms per liter of tea (6 μg/L) (Mulder et al., 2015). The European Food and Safety Authority calculated a margin of exposure to 237 g/kg body weight per day for pyrrolizidine alkaloids and their *N*-oxides (Knutsen et al., 2017; Kaltner et al., 2020). No genotoxicity was detected as pink fluorescent zone for the 68 botanical extracts applied at 400 µg/band (Figures 2–4O). This bioassay was repeated to prove for the absence of genotoxic effects at a 2.5-fold (Supplementary Figure S6) and 12.5-fold higher amount applied, which latter at 5 mg/band was closest to overloading the chromatographic system (Supplementary Figure S7).

#### 3.2.8 Compounds Inhibiting α-Amylase

In the context of hypoglycemic drugs from nature, not only the mentioned α- and β-glucosidase inhibitors, but also α-amylase inhibitors play a role in ethnopharmacological remedies with less toxic side effects. Hyperglycemic blood levels could be reduced by inhibiting α-amylases of the saliva and pancreas. The inhibition reduces the cleavage of starch into oligo- and disaccharides, and so the release of glucose molecules absorbed postprandially into the blood. Plant-derived flavones as luteolin present in celery, parsley, broccoli, carrot, peppers, cabbage, and apple peel can inhibit α-amylase (Bai et al., 2019). HPTLC coupled to α-amylase assay (Figures 2–4P) showed only a few inhibiting signals in white horehound (no. 2, *hR*_F_ 43, 83, 91, and 93), artichoke (no. 4, *hR*_F_ 94), fenugreek (no. 6, *hR*_F_ 81), ginkgo (no. 14, *hR*_F_ 46), licorice (no. 55, *hR*_F_ 59 and 68), and lemon peel (no. 67, *hR*_F_ 93 and 95). An α-amylase inhibitory activity has already been described for artichoke extracts from bracts (Turkiewicz et al., 2019), but it was not assigned to any single compound. In *Ginkgo biloba* extracts the compound sciadopitysin (C_33_H_24_O_10_) was found to potentially inhibit α-amylase (Petersen et al., 2019).

### 3.3 Assigning Bioactivity to Single Compounds

In previous work, the samples were applied twice (as two sets). After plate cut, the assay was performed on one plate part, and the positions of bioactive zones were analogously marked on the other plate part for zone elution and transfer into the MS (Krüger et al., 2017). The challenge of this parallel handling was the accurate positioning of the elution head on the zone. Instead, the elution directly from the bioassay plate would avoid any possible mismatch. In addition, the orthogonal HPLC separation would solve coelution. Hence, zones of interest were heart-cut eluted directly out of the bioassay plate and transferred through an online desalting device to RP-HPLC−DAD−HESI-MS. This reduced the interfering salts and nutrients from the bioassay, separated possibly coeluting substances via the orthogonal chromatographic system, and added value via spectral and mass spectrometric data (Schreiner and Morlock, 2021). Through this straightforward comprehensive workflow, the 60 most bioactive compounds in the botanicals were assigned (Table 2). Several plant extracts out of the 68 botanicals contained multipotent compounds which were active in different assays. Among striking botanicals, such as galangal (no. 13), ginkgo (no. 14), yerba mate green (no. 37), orange peel (no. 41), licorice (no. 55), or Siberian ginseng (no. 56), four botanicals were exemplarily highlighted to demonstrate the targeted assignment of bioactive compounds (orange peel, Figure 7; licorice, Figure 8; galangal, Figure 9; yerba mate green, Figure 10). By transferring residual bioassay salts, different ion species were formed for a molecule, which was found to be helpful as it confirmed the assignment made several times. The adduct [M+62]^−^ has not been described in HPTLC-MS literature so far, but appeared frequently during this study (Figures 7C,D, Figures 8C,D, Figures 11A,C). It was proposed to be the nitrate adduct. Nitrate is taken up by the root and transported via the xylem to leaves, shoots, and grains. If too much nitrate is available in the short term, which is the case as it is discussed as a global environmental challenge (Zhang et al., 2021), it passes through the cytoplasm into the vacuoles to be stored and thus can be found in botanicals (Dechorgnat et al., 2011). Consequently, it may also appear as a pronounced adduct in the mass spectrum.

#### 3.3.1 Hormonal Antagonists in Orange Peel (No. 41)

For orange peel, manifold positive responses across the different assays were observed at *hR*_F_ 34 (Figure 7A, marked*). Using the pYEAS bioautogram, this zone was heart-cut eluted directly to RP-HPLC−DAD−HESI-MS. In the DAD chromatogram, three compound peaks were evident in the range of RT 6.87–7.07 min, apart from the 4-methylumbelliferone background signal at RT 5.71 min (Figure 7B). The corresponding mass spectral data led to the tentative identification of naringin (Figure 7C), hesperidin (Figure 7D), and meranzin (Figure 7E, coeluting with an unknown marked blue) and is discussed as follows. The total ion current (TIC) peak at RT 6.87 min revealed mass signals in the positive ionization mode at *m/z* 581 [M + H]^+^, 603 [M + Na]^+^, and 619 [M + K]^+^. Corresponding mass signals in the negative ion mode were detected at *m/z* 579 [M−H]^−^, 615 [M + Cl]^−^, and 642 [M + NO_3_]^−^ (Figure 7C). The resulting neutral mass of 580 Da together with the absorption spectrum and maximal wavelengths (λ_max_ = 220 and 284 nm) suggested naringin, which was confirmed via co-chromatography against a bought standard (Supplementary Figure S8E). The extracted mass spectra of the TIC peak at RT 6.99 min showed positive ions at *m/z* 611 [M + H]^+^, 633 [M + Na]^+^, and 649 [M + K]^+^ and negative ions at *m/z* 609 [M−H]^−^, 645 [M + Cl]^−^, and 672 [M + NO_3_]^−^ (Figure 7D). The absorption spectrum revealed a maximum wavelength at 284 nm. Based on this data, hesperidin was assumed. The third peak at RT 7.06 min had a major response in TIC-HESI^+^ and a minor response in TIC-HESI^−^ and DAD chromatograms. The UV absorbance spectrum and respective mass spectra suggested two different constituents. The HESI^+^ mass signals at *m/z* 261 [M1+H]^+^ and 278 [M1+NH_4_]^+^ potentially belong to the coumarin derivate meranzin (Figure 7E). The HESI^+^ mass signals at *m/z* 441 [M2+H]^+^, 458 [M2+NH_4_]^+^, 463 [M2+Na]^+^, and 479 [M2+K]^+^ and corresponding HESI^−^ mass signals at *m/z* 475 [M2+Cl]^−^, 485 [M2+HCOO]^−^, and 499 [M2+H_3_C-COO]^−^ were assigned to a component with the neutral mass of 440 Da, which is not yet known in orange peel. The UV maxima at 258 and 325 nm provided additional evidence for meranzin (Fan et al., 2012). Other UV maxima at 223 and 288 nm were possibly induced by the unknown.

*Citrus sinensis* is rich in beneficial secondary metabolites and therefore traditionally used in the treatment of gastrointestinal malfunction, diseases of the upper respiratory tracts, or menstrual disorders (Favela-Hernández et al., 2016). Both naringin and hesperidin have been shown to bind to the antagonist pocket (3ERT) of hERα, and thereby cause an antiestrogenic effect (Puranik et al., 2019). This effect was verified for both via co-chromatography of standards and samples (Supplementary Figure S8E, Supplementary Table S1), whereby an estrogenic activity was not observed for the applied amount (4 µg/band). Furthermore, naringin was reported to slightly bind to the androgen receptor (Fang et al., 2003). The pronounced antiandrogenic effects observed in the pYAAS bioautogram confirmed this (Figure 3M, no. 41). Tyrosinase activity attributed to naringin (Itoh et al., 2009) and hesperidin (Zhang et al., 2007) has already been demonstrated. The only bioactivity reported for meranzin was no effect (Smyth et al., 2009) or a minor (Rosselli et al., 2007) antibacterial effect against *B. subtilis*. After NP-HPTLC–FLD comparison to a standard, meranzin was located at *hR*_F_ 99 (Supplementary Figure S8G), where antibacterial activity against *B. subtilis* and *A. fischeri* was detected in orange peel (no. 41). Hence, the assumption that meranzin was co-eluting with hesperidin and naringin at *hR*_F_ 34 was discarded.

#### 3.3.2 Enzyme Inhibition and Endocrine Activity in Licorice (No. 55) and Galangal (No. 13)

In licorice (no. 55) a few bioactive analytes were found in the zone at *hR*_F_ 31 with antibacterial, tyrosinase, and β-glucuronidase inhibitory, antidiabetic, and endocrine-antagonistic properties (Figure 8A, marked*). These results illustrate the diverse pharmacological activities of this root, which has long been used in traditional medicine as remedy to treat gastrointestinal problems (β-glucuronidase inhibition) and respiratory infections (antibacterial activity). Moreover, *Glycyrrhiza glabra* extracts were subject of many pharmacological studies showing neuroprotective, antimicrobial, estrogenic and skin-whitening activity (Pastorino et al., 2018). The second chromatography produced five individual signals with pure mass spectra (Figures 8B–E). The mass spectral data extracted from the peaks at RT 6.52/6.63 min (colored blue) were identical, indicating a single analyte in different configurations (Figure 8C). The ESI^−^ signals at *m/z* 549 [M−H]^−^, 585 [M + Cl]^−^, and 612 [M + NO_3_]^−^ were correlated to the highly plant-specific liquiritin apioside with a neutral mass of 550 Da. In positive ion mode, the signals at *m/z* 573 and 589 were identified as sodium and potassium adducts, respectively. The ESI^+^ mass signals at *m/z* 257 [M−C_11_H_18_O_9_+H]^+^ and 419 [M−C_5_H_8_O_4_+H]^+^ could be assigned to fragments with a loss of carbohydrates. Additionally, the experimental UV absorbance spectra were consistent with the ones of liquiritin apioside found in literature (Wong et al., 2018). The isomeric isoliquiritin apioside probably caused the second peak at RT 6.63 min. ESI^+^ mass signals at RT 7.10 min were *m/z* 563 [M + H]^+^, 585 [M + Na]^+,^ and 601 [M + K]^+^ (Figure 8D). (Iso)liquiritin apioside was found to inhibit capsaicin-induced cough, confirming its traditional use (Pastorino et al., 2018). In negative ion mode, signals were detected at *m/z* 561 [M−H]^−^, 597 [M + Cl]^−^, 621 [M + H_3_C-COO]^−^, and 624 [M + NO_3_]^−^. The less abundant DAD signal showed a UV absorption maximum at 250 nm. The spectral data could indicate glycyroside found in licorice root extracts (Montero et al., 2016), which is not known for any bioactivity. The third signal at RT 7.86 min (colored orange) was identified as glycyrrhizic acid against a standard (Supplementary Figure S8E). The ESI^−^ and ESI^+^ ions at m/z 821 [M−H]^−^, 843 [M−2H + Na]^−^, 859 [M−2H + K]^−^, 845 [M + H]^+^, and 861 [M + K]^+^ originated from the neutral molecule of 822 Da. Lacking a π-electron system and conjugated double bonds, the UV absorbance spectrum showed background absorbance maxima at 251 nm. Glycyrrhizic acid was reported to have anti-inflammatory effects (Yu et al., 2015) similar to those of glucocorticoids (Pastorino et al., 2018), antitussic activity through increased tracheal mucus secretion (Sharma et al., 2018), and neuroprotective (Kao et al., 2009) properties.

Galangal extract (no. 13) responded in almost all assays at *hR*_F_ 99 (Figure 9A, marked*). After separating this zone with RP-HPLC-DAD-HESI-MS, multiple signals were observed (Figure 9B). The spectral details and tentative identifications are listed in the table below (Figure 9C). Traditionally, *Alpinia officinarum* is used against cold, which antibacterial effects were confirmed by the Gram-negative *A. fischeri* and Gram-positive *B. subtilis* bioassays. Other traditional applications were described for gynecological disorders, diabetes treatments, and skin washing (Abubakar et al., 2018). All these bioactivities were verified by the respective assays. To assign the bioactivity to one single component out of the coeluting substances from HPTLC, fractionation after column separation is necessary. The fractions can be applied again on a new plate followed by EDA and MS characterization. Alternatively, the mobile phase for planar chromatography has to be optimized in order to separate the previously coeluting substances during HPTLC analysis.

#### 3.3.3 Separating Multipotent Isomers in Yerba Mate Green (No. 37) via 8D-Hyphenation

Biologically active isomers were also separated and detected, shown for example in yerba mate green (no. 37). A multipotent bioactive compound zone (*hR*_F_ 40) was observed in AChE, tyrosinase, and α-/β-glucosidase inhibition autograms (Figure 10A, marked*). This zone was separated into three distinct signals via RP-HPLC (Figure 10B), all providing the same absorbance and mass spectra (Figure 10D) and assigned to chlorogenic acid isomers, which differed only in quinic acid positioning (Figure 10C). The most common isomers of mono-caffeoylquinic acid are 3-*O*-caffeoylquinic acid (neochlorogenic acid), 4-*O*-caffeoylquinic acid (cryptochlorogenic acid), and 5-*O*-caffeoylquinic acid (5-QCA, chlorogenic acid). The mass signals at *m/z* 353 [M−H]^−^ and 707 [2M−H]^−^ in the negative ionization mode and at *m/z* 355 [M + H]^+^, 377 [M + Na]^+^, 393 [M + K]^+^ and 747 [2M + K]^+^ in the positive ionization mode matched to the neutral mass of mono-caffeoylquinic acids of 354 Da. The UV absorbance spectra with maxima at 218 and 325 nm that we obtained were also consistent with literature (Velkoska-Markovska et al., 2020). While chlorogenic acids are reported to have antioxidant (Huang et al., 2017), anti-inflammatory (Willems et al., 2016; Huang et al., 2017), and anti-HIV (Tamayose et al., 2019) properties, this screening revealed even more bioactive potential for these phenolics. For instance, the assigned bioactive compound zone also showed anti-Alzheimer, antidiabetic, and skin-whitening effects in this study. Co-chromatography against standards (Supplementary Figure S8A,B and D, Supplementary Table S1) confirmed that these beneficial health effects of yerba mate green (no. 37, Figure 10) come from the mono-caffeoylquinic acids. The various effects of *Ilex paraguariensis* traditionally consumed as herbal beverage qualify this botancial for its new role as functional food (Cardozo Junior and Morand, 2016).

#### 3.3.4 Universal Potential of 8D-Hyphenation

The characterization of additional multipotent bioactive zones, partially plant-specific, is shown in Figure 11. Artichoke (no. 4) showed bioactivity at *hR*_F_ 42 in the tyrosinase and α-/β-glucosidase inhibition assays. Transferring this zone to RP-HPLC-DAD-HESI-MS provided five signals (Table 2), one of which is specific for artichoke. At RT 6.30 min, cynarascoloside C was assumed (Figure 11A). Spectral data showed no UV absorbance, referring to structural properties of cynarascoloside C which possess neither a π-electron system nor conjugated double bonds. The mass signals at *m/z* 444 [M + NH_4_]^+^, 449 [M + Na]^+^, 465 [M + K]^+^, 461 [M + Cl]^−^, 471 [M + HCOO]^−^, 485 [M + H_3_C-COO]^−^, and 488 [M + NO_3_]^−^ indicated a neutral mass of 426 Da. Cynarascoloside C was not suspected to have bioactive effects. The bioactivity was probably attributed to the coeluting derivates of chlorogenic acid as in yerba mate green (no. 37, Figure 10). Nevertheless, *Cynara scolymus* has been used since the 4th century B.C. as medicinal product due to its health benefits and bioactive constituents, responsible for the hypoglycemic, anti-inflammatory, antimicrobial, and antioxidant properties (Turkiewicz et al., 2019).

*Eucalyptus* (no. 9) is known for its beneficial health properties against infections of the upper respiratory tracts or as antiseptic and is therefore widely used in the pharmaceutical industry (Hasegawa et al., 2008; Ács et al., 2018). The species *Eucalyptus* is closely associated with herbal medicine and traditional health care in various human cultures. Its leaf extracts are administered to fight against cold and cough, bacterial infections, high blood glucose levels, and to boost the immune system and skin health (Salehi et al., 2019). Planar bioanalytical screening confirmed most of those traditional uses, showing many positive responses through the assays in a wide *hR*_F_ range (Figure 11B). Focusing on *hR*_F_ 57, highly plant-specific eucaglobulin was identified with UV (λ_max_ = 222 and 261 nm) and mass spectral data (Boulekbache-Makhlouf et al., 2010). Both the positive ion species at *m/z* 521 [M + Na]^+^ and 537 [M + K]^+^, and the deprotonated molecule [M−H]^−^ confirmed this suspicion. Anti-melanogenesis activity was attributed to eucaglobulin (Hasegawa et al., 2008) and proved with a planar tyrosinase bioassay. Extracts of eucalyptus fruits were demonstrated to have antibacterial effects against *B. subtilis* (Boulekbache-Makhlouf et al., 2013), which so far have not been directly correlated to the monoterpene conjugate eucaglobulin. The anti-cholinesterase activity was only described for the whole methanolic extract of *Eucalyptus globulus* (Amat-ur-Rasool et al., 2020) but not directly correlated to eucaglobulin.

Extracts of *Ginkgo biloba* seeds and leaves were applied as herbal remedies all over the globe. Originating from traditional Chinese medicine, it is used to treat bacterial skin diseases, cognitive decline (Chassagne et al., 2019), and cardiovascular diseases (Shu et al., 2018). Affirming the ethnopharmacological usage, EDA of ginkgo leaf extract (no.14) demonstrated a variety of bioactivity at *hR*_F_ 93, *i.e.,* antibacterial and antidiabetic effects, as well as β-glucuronidase and tyrosinase inhibition, and antiandrogenic activity (Figure 11C). RP-HPLC-DAD-HESI-MS analysis revealed the three ginkgolides A–C incorporated in this bioactive zone. Ginkgolide C eluted earlier from RP column (RT 6.42 min). Despite the second chromatography, ginkgolides A and B were not separated (both RT 6.86 min) and were thus detected in the same UV and mass spectra. Structurally they only differ in one additional hydroxy group of ginkgolide B. The high abundance of different ion species describing the two compounds is listed in Table 2 and displayed in Figure 11C. The wide bioactive spectrum of these two diterpenes was confirmed against standards (Supplementary Figure S8B, Supplementary Table S1).

As an alternative to coffee, the leaves of *Ilex paraguariensis* are widely consumed as beverage in Latin America. Due to its chemical composition, mainly alkaloids and polyphenols, yerba mate exhibits many bioactive effects, *e.g.,* antibacterial, cardiovascular-protective, neuroprotective, and antidiabetic activities (Gan et al., 2018). In yerba mate green (no. 37), a multipotent bioactive analyte zone was detected at *hR*_F_ 20 (Figure 11D). Showing signals at *m/z* 609 [M−H]^−^ in ESI^−^ and at *m/z* 611 [M + H]^+^, 633 [M + Na]^+^, and 649 [M + K]^+^ in ESI^+^ at an RT of 6.84 min, the flavonoid rutin was assumed. Its antibacterial (Orhan et al., 2010) potential against *B. subtilis*, anti-tyrosinase activity (Kishore et al., 2018), and glucosidase inhibition (Li et al., 2009) have already been demonstrated. The assumption was verified against a standard (Supplementary Figure S8A and D, Supplementary Table S1).

*Salvia rosmarinus*, predominantly growing in Mediterranean regions, is independently used in Mexican and Spanish ethnopharmacology. As medicinal plant, it was used to fight bacterial skin diseases, colds, intestinal parasites, and headaches (Heinrich et al., 2006). A very broad spectrum of bioactivities was confirmed by the presented study. The comprehensive NP-HPTLC-EDA-heart-cut RP-HPLC-DAD-HESI-MS analysis of rosemary (no. 46) showed antibacterial activity in the *A. fischeri* bioassay and antidiabetic properties in the β-glucosidase assay at *hR*_F_ 25. Several ions at *m/z* 387 [M−H]^−^, 406 [M + NH_4_]^+^, 411 [M + Na]^+^, and 427 [M + K]^+^ in positive and negative ion mode were correlated to the neutral molecular weight of 388 Da (Figure 11E). The mass spectrometric data substantiate the suspicion that this bioactivity is related to medioresinol. In rosemary extracts, various bioactive compounds have been described. Carnosic acid and carnosol are known to inhibit the growth of human cancer cell lines and operate as anti-inflammatory agents (Bai et al., 2010; Wang et al., 2018) or antioxidants (Loussouarn et al., 2017). Rosmarinic acid and rosmanol were found to have antioxidant potential (Vallverdú-Queralt et al., 2014), but to our knowledge, there is no bioactivity reported for medioresinol.

Many more examples could be explained in detail. All have in common that the effect profiles we obtained explain why consuming green tea and using fresh herbs and spices as seasoning can reduce the risk of diabetes by inhibiting α- and β-glucosidases, why healthy nutrition can protect the intestinal flora from severe impairment caused by β-glucuronidases from *Enterobacteriaceae*, why plant-derived cosmetics can reduce skin abnormalities via its tyrosinase-inhibiting potential, and why Alzheimer’s disease can be prevented by daily intake of chlorogenic acid or rosmarinic acid from herbs such as artichoke, lemon balm, peppermint, thyme and rosemary, and so on. The wealth of effect information obtained inspires the mind and could fuel further studies. An enormous diversity in bioactivity was revealed in the effect-profiles of the 68 botanicals, contributing to human health by drug-like properties. It clearly shows the potential and spectrum of nature as basis for alternative medicines.

## 4 Conclusion

Since the major part, and especially, the important active part of natural food and traditional medicines is presently not under analytical control, a paradigm shift from quality control based on marker compounds to effect profiles is postulated for plant-based samples. Considering the global production chain, whose influences on the product cannot be controlled, at least entries or changes regarding the effect should be kept under control. The Chemical Abstracts database (www.cas.org) contains over 190 million chemicals, and thousands of compounds are added daily. There is of little help, if we can measure some thousands of them with ever lower limits of determination. By doing so, this does not come close to doing justice to the complexity of plant extracts, nor to the metabolic networking of natural processes, nor to the contamination-prone global production chain. The implementation of effect-directed profiles would subtantially improve quality control, ensure the expected activites and detect unexpected activities. Even small amounts of compounds can be highly active. Disruptive thinking is essential to better control our natural food and traditional medicines. Sophisticated instrumentation does not solve pressing challenges, but combining orthogonal areas does. The complexity of plant extracts requires modern non-target methods that combine chromatography with effect-directed assays to prioritize active compounds that show an effect and thus require utmost attention. This combination is indispensable for routine quality control to prioritize substances among the thousands of individual compounds in a plant extract. The modular NP-HPTLC−UV/Vis/FLD−EDA-heart-cut RP-HPLC−DAD−HESI-MS coupling can be used in routine and makes it easy to recognize the essence. It is said that a picture is worth a thousand words, but the effect image is worth even more. It visualizes the impressive power of nature to supply the body with important building blocks (multipotent chemicals). Several innovations were demonstrated. The pYES equipped with the hERβ or in combination with the simulated S9 metabolization were applied for the first time. In the antagonistic hormonal assays, the proof for false-positive results was newly included. Synergistic effects were revealed in the hormonal effect-profiles for the first time. The developed workflows can be transferred to any other assays or samples. The array of effect-directed profiles clearly showed that natural food has the power to contribute to our homeostasis in various effective ways. The 1,292 profiles (68 samples x 19 detections) obtained within a few weeks showed the versatility of the activity potential of natural food. Artificial intelligence could help evaluate the wealth of information obtained. Exemplarily, the 60 most bioactive components were identified as proof of principle. The developed non-targeted effect-directed hyphenation highlights the advantages of analytical speed, efficiency, and economy. First, the samples were freed from the interfering matrix via planar chromatographic separation. Secondly, the focus was exclusively laid on bioactive compounds, providing targeted characterization. Calculated per sample, the robust profiling takes 3–15 min and costs 0.5–1 Euro, depending on incubation time and material consumption, respectively. One current limitation is the low resolution of the single quadrupole MS instrument. Potential drug candidates were only tentatively assigned and confirmed in an additional run against standards. At the same time, this limitation can be an opportunity for further research. Upgrading the MS instrument to a high-resolution MS with fractionation possibility enables unambiguous assignment of molecular formulas and structure elucidation through fragmentation. This makes effect profiling even more attractive for routine analysis.

## Data Availability

The original contributions presented in the study are included in the article/Supplementary Material, further inquiries can be directed to the corresponding author.
