# Supplementary material for: Is Our Natural Food Our Homeostasis? Array of a Thousand Effect-Directed Profiles of 68 Herbs and Spices
Source: Front Pharmacol. 2021 Dec 9;12:755941. doi: 10.3389/fphar.2021.755941 (PMC8696259; doi:10.3389/fphar.2021.755941)
Supplement: Supplementary file 1 [file DataSheet1.docx]

Supplementary Material


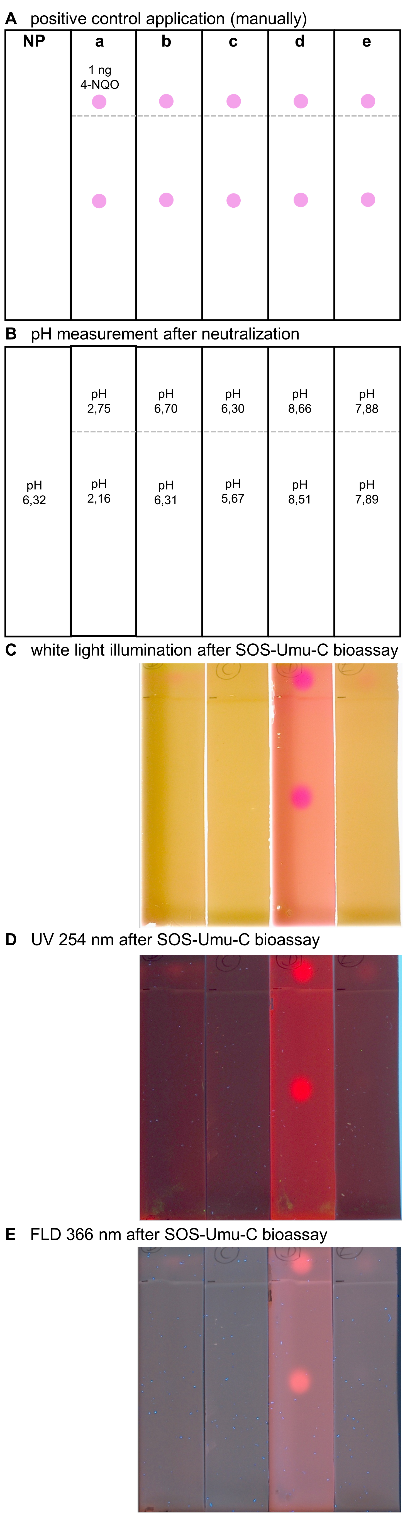


**Supplementary Figure 1.** NP-HPTLC–SOS-Umu-C neutralization study. An empty HPTLC plate silica gel 60 was developed with ethyl acetate – toluene – formic acid – water (16:4:3:2, *V/V/V/V*), up to 70 mm and cut into 5 pieces (A). 4-nitroquinoline 1-oxide (1 µL, 1 ng/µL) was applied within and above the solvent front. The pH of differently treated plate pieces was measured with a contact electrode (B): untreated plate (NP), not neutralized (a), immersion (3.5 cm/s, 2 s) in citrate buffer pH 12 (b), spraying (yellow nozzle, level 2) 2.8 mL citrate buffer pH 12 (c), immersion (3.5 cm/s, 2 s) in 2.5% sodium bicarbonate buffer pH 8 (d), 20 min ammonia vapor (e). After the SOS-Umu-C bioassay, plates were detected at white light illumination (C), UV 254 nm (D) and FLD 366 nm (E).


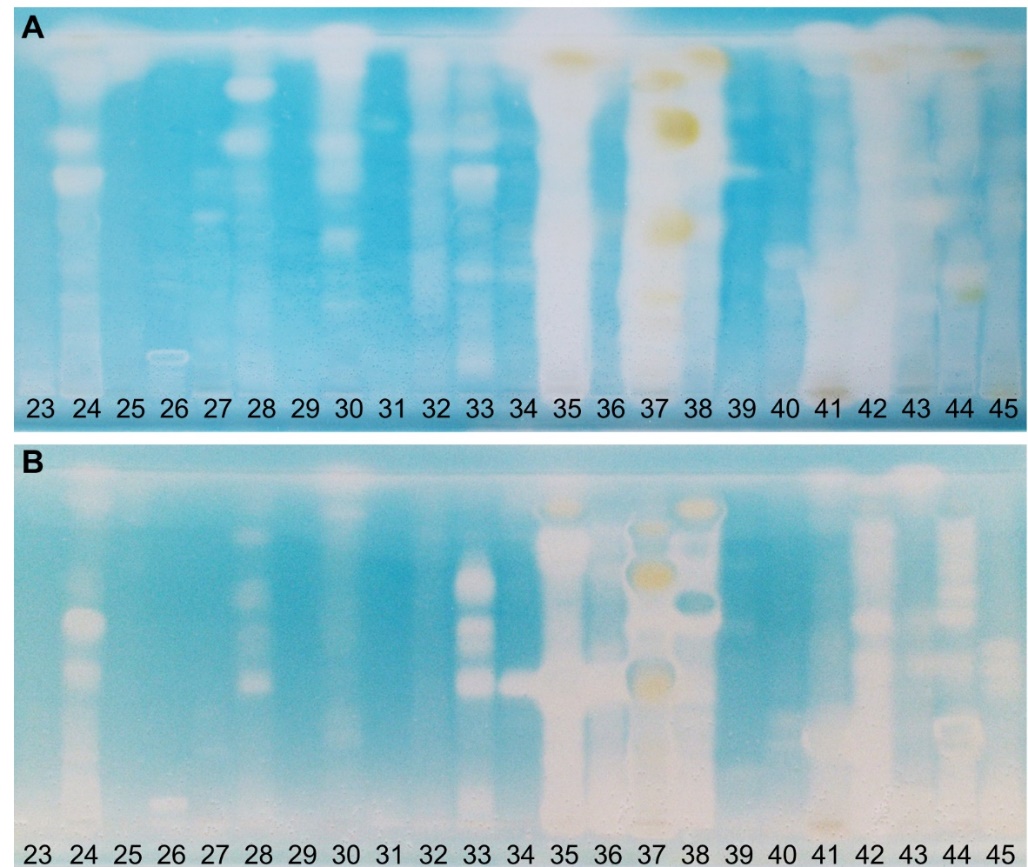


Supplementary Figure 2. NP-HPTLC–β-glucosidase inhibition profiles of the plant extracts nos. 23–45 (assignments in Table 1) applied as 4 µL/band (A) and 1 µL/band (B) on HPTLC plate silica gel 60 F_254_ MS-grade, developed with ethyl acetate – toluene – formic acid – water (16:4:3:2, *V/V/V/V*), up to 70 mm, detected at white light illumination after the β-glucuronidase inhibition assay.


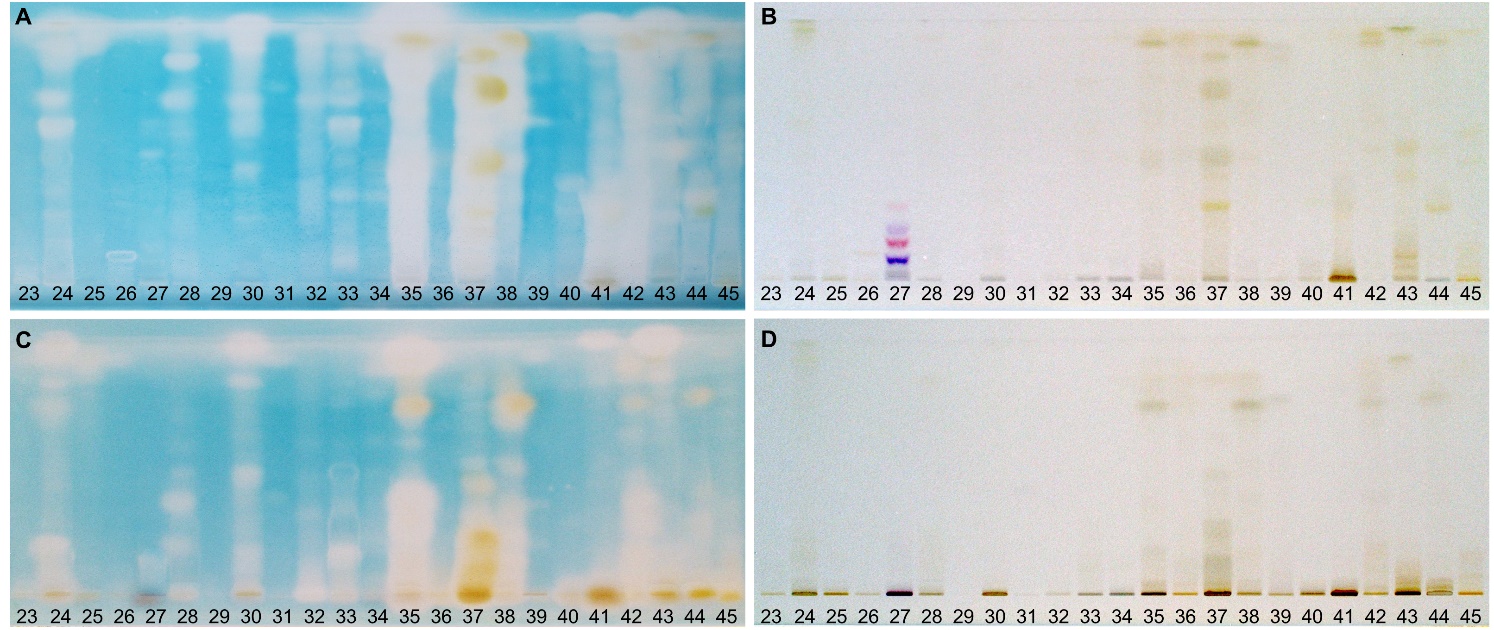


Supplementary Figure 3. NP-HPTLC–β-glucosidase inhibition profiles of the plant extracts nos. 23–45 (4 µL/band, assignments in Table 1) applied on HPTLC plate silica gel 60 F_254_ MS-grade, developed with ethyl acetate – toluene – formic acid – water (16:4:3:2, *V/V/V/V*) (A, B) or ethyl acetate – toluene – formic acid (10:4:1, *V/V/V*) (C, D) up to 70 mm, detected at white light illumination before (B, D) and after (A, C) the β-glucuronidase inhibition assay.


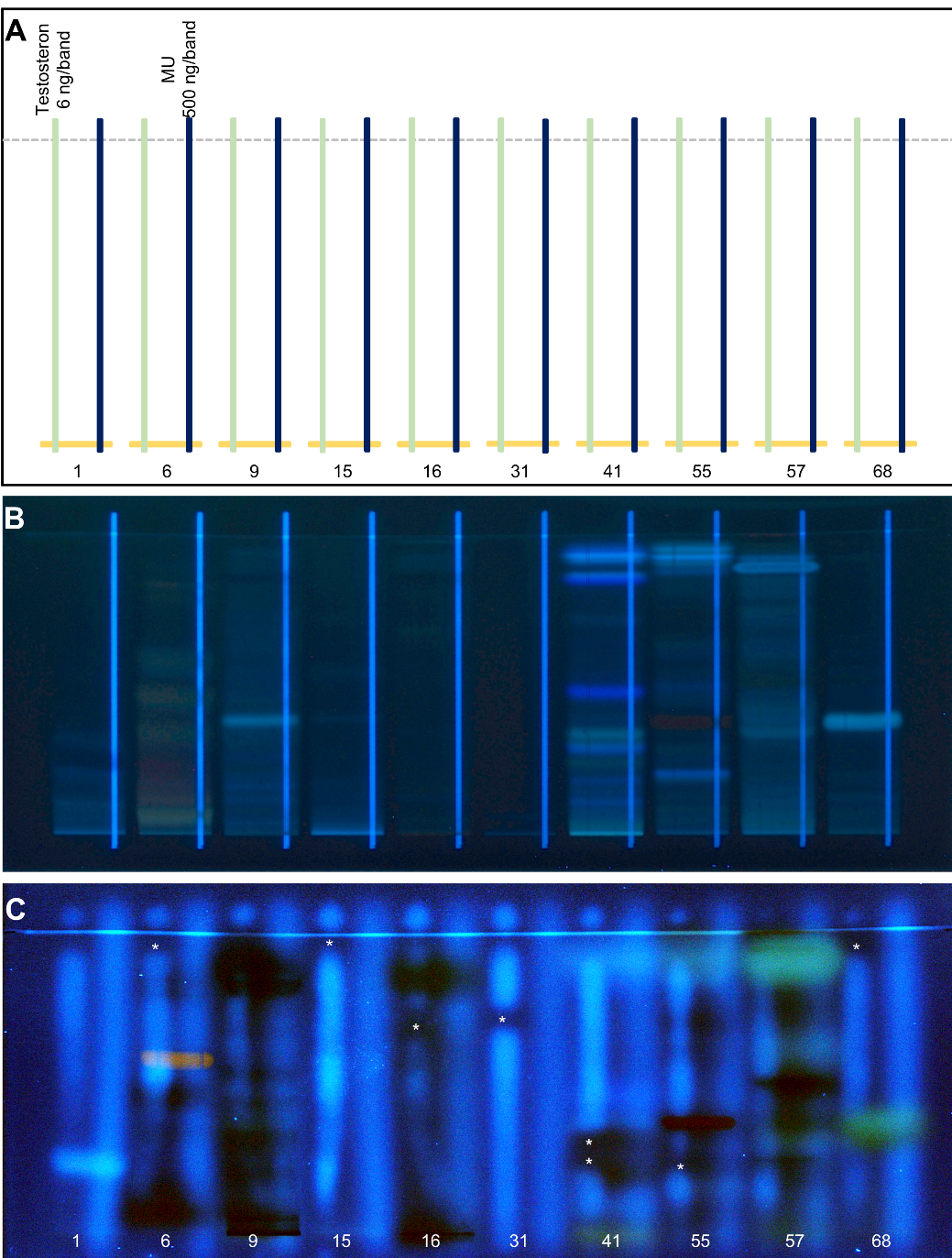


Supplementary Figure 4. Exclusion of false-positive results in NP-HPTLC-pYAAS bioassay. The samples (assignments in Table 1) assumed to have antiandrogen-like effects were investigated for false positive results by application as 15 mm bands (5 µL) and development up to 70 mm with ethyl acetate – toluene – formic acid – water (16:4:3:2, *V/V/V/V*). 4‑Methylumbelliferone (500 ng/band) and testosterone (6 ng/band) were applied as an overlapping 1 mm × 70 mm area on each track according to plate design (A). Application was monitored at FLD 366 nm (B). Comparison of testosterone and 4‑methylumbelliferone fluorescence deletion after performing the pYAAS bioassay documented at FLD 366 nm (C). Antiandrogen-like zones only delete fluorescence in overlapping testosterone area (marked*).


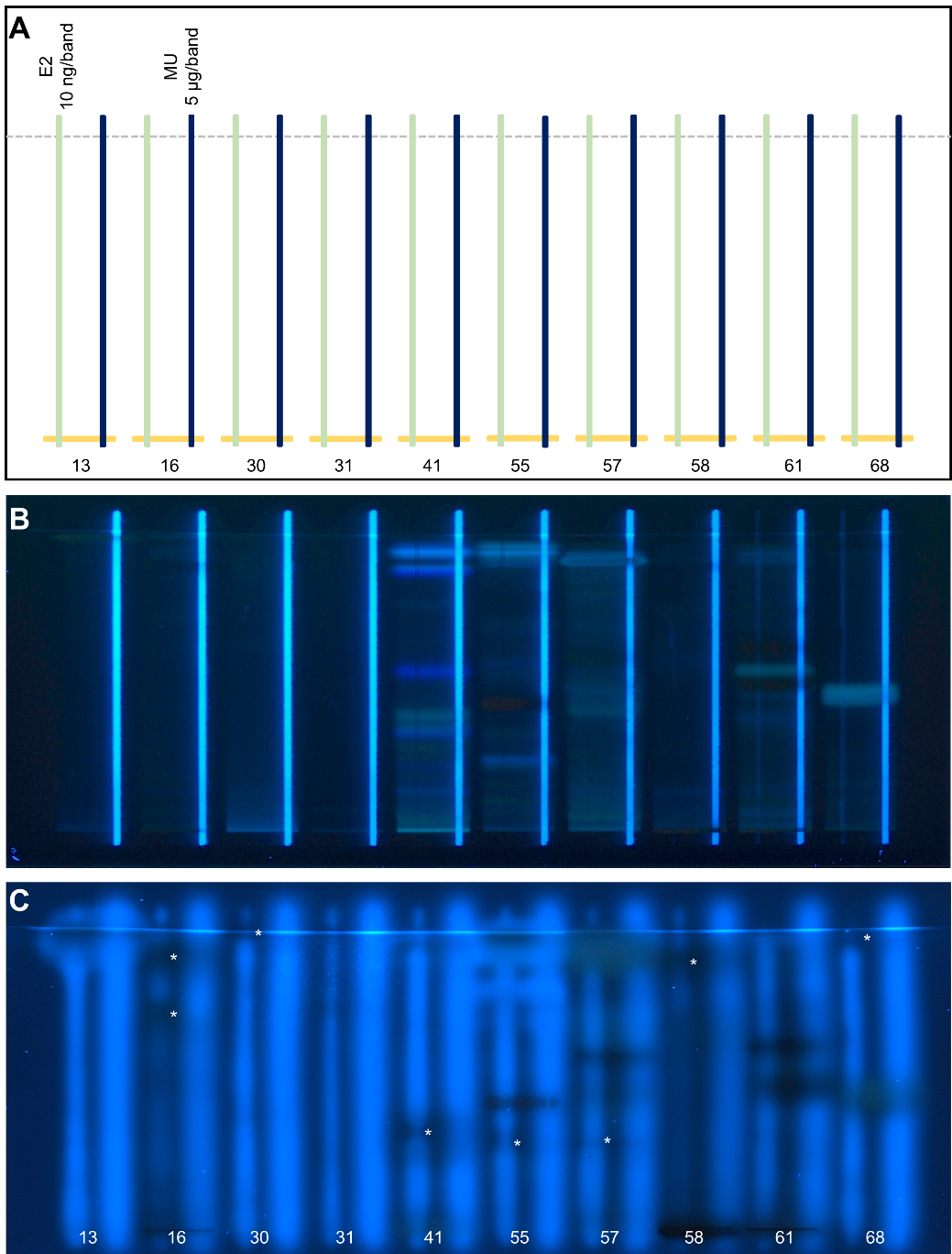


Supplementary Figure 5. First report of the NP-HPTLC-pYAES bioassay with the integrated exclusion of false-positive results. Selected botanicals (nos. 13−68 assigned in Table 1) assumed to have antiestrogen-like effects were proven for truely anti-estrogenic or false positive results according to the plate design (A): application each as 15-mm band (5 µL), development up to 70 mm with ethyl acetate – toluene – formic acid – water (16:4:3:2, *V/V/V/V*) and detection at FLD 366 nm (B); 4‑methylumbelliferone (5 µg/band) and 17-β-estradiol (10 ng/band) were oversprayed as 1 mm × 70 mm area on each separated track, followed by the bioassay application and documentation at FLD 366 nm (C). Comparing the 17-β-estradiol and 4‑methylumbelliferone fluorescence reduction reveals truely antiestrogen-like zones which only reduce the fluorescence in the 17-β-estradiol area (marked*).


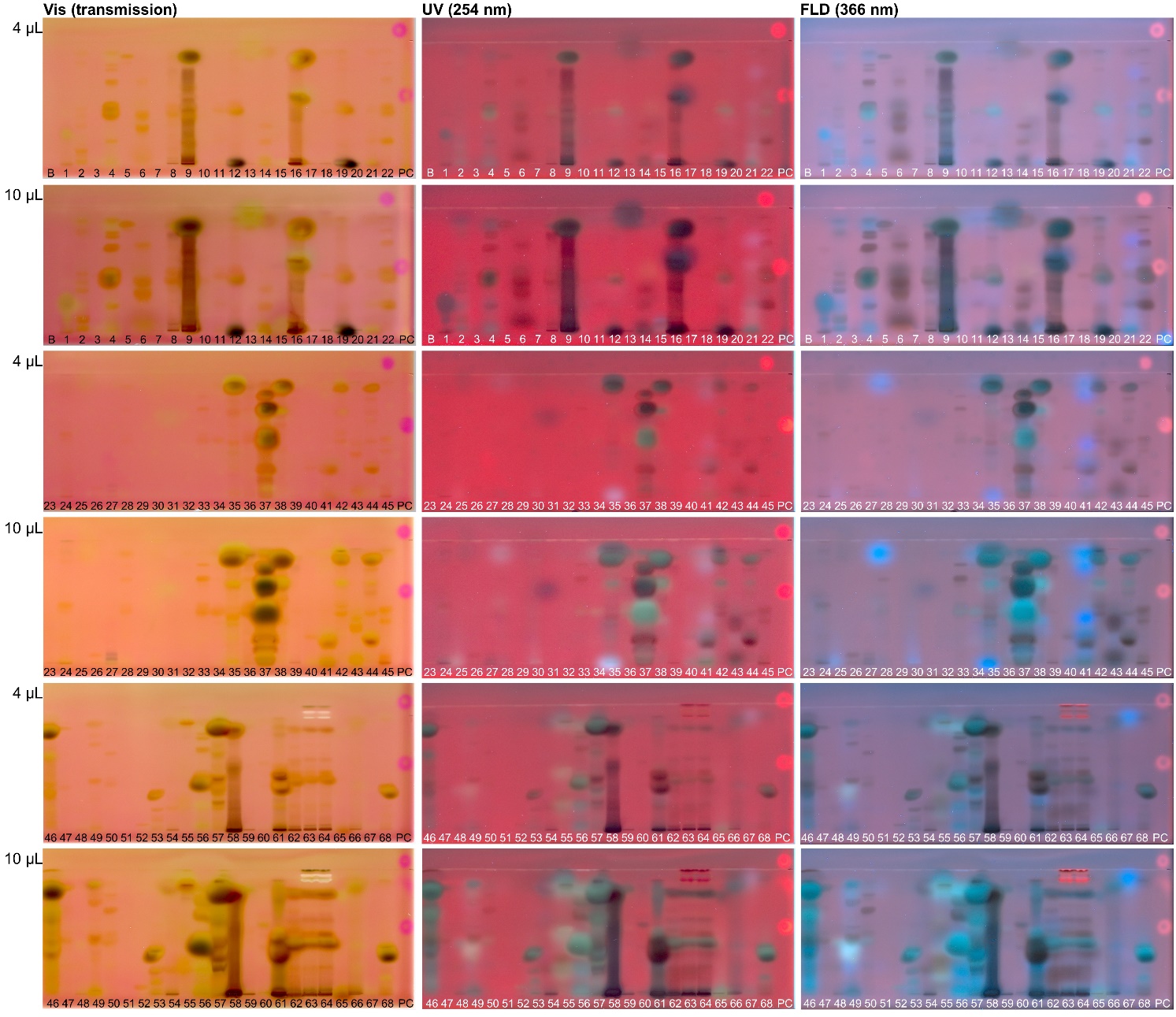


Supplementary Figure 6. NP-HPTLC–SOS-Umu-C profiles of the plant extract nos. 1–68 (4 or 10 µL/band respectively; assignments in Table 1; solvent blank B for comparison) and positive control (4-nitroquinoline 1-oxide 1 µL, 1 ng/µL) on HPTLC plate silica gel 60 with ethyl acetate – toluene – formic acid – water (16:4:3:2, *V/V/V/V*), up to 70 mm, detected at white light illumination (Vis), UV 254 nm and FLD 366 nm after the SOS-Umu-C bioassay.


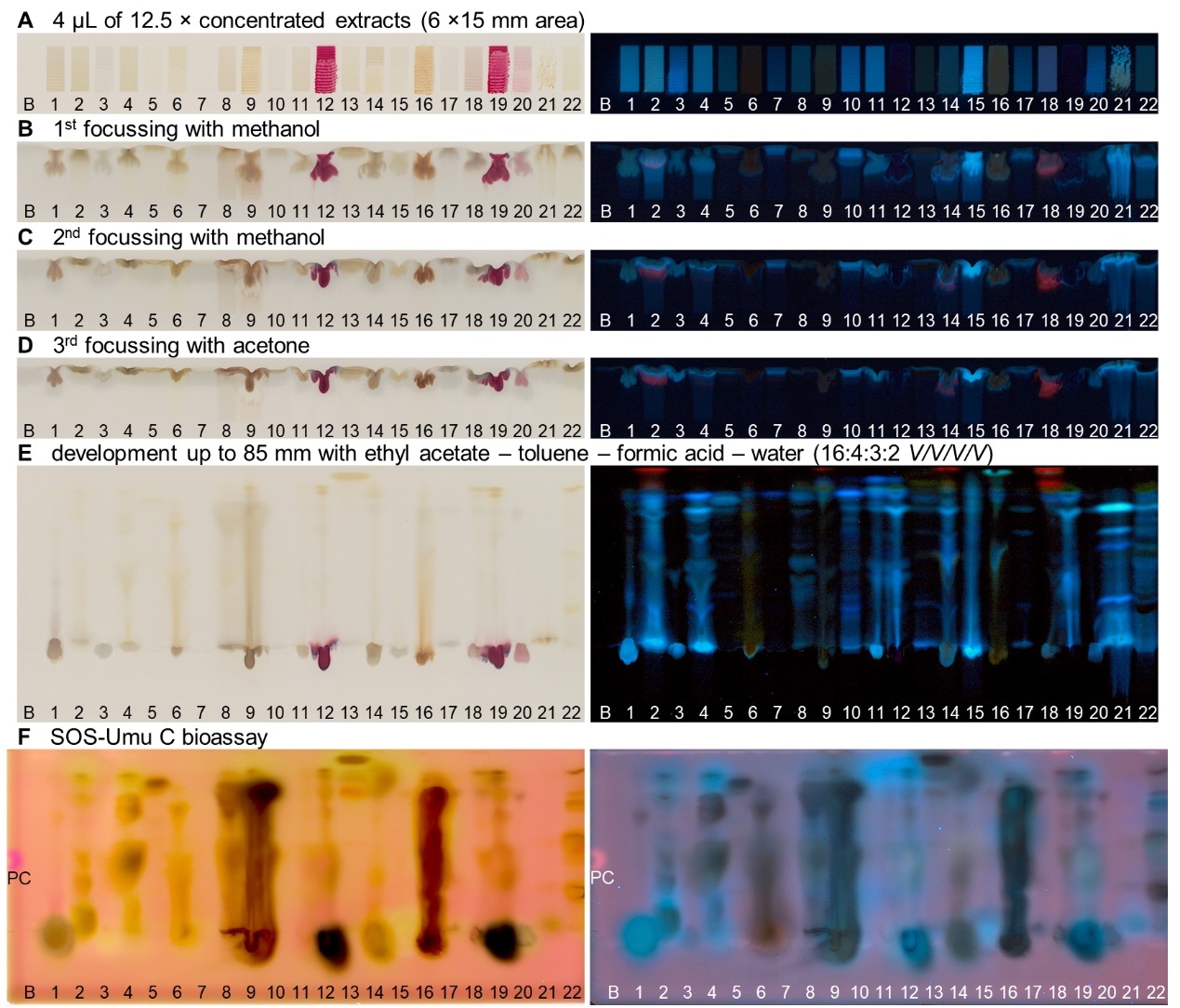


Supplementary Figure 7. NP-HPTLC–SOS-Umu-C profiles of the plant extracts nos. 1–22. Area application (6 mm × 15 mm) of the 12.5‑fold concentrated botanicals (4 µL/area respectively, assignments in Table 1; solvent blank B for comparison) and positive control (1 µL, 1 ng/µL 4‑nitroquinoline 1-oxide) on HPTLC plate silica gel 60 (A). Focusing of the areas with methanol (B, C) and acetone (D) to 25 mm and development with ethyl acetate – toluene – formic acid – water (16:4:3:2, *V/V/V/V*) up to 85 mm (E), detected at white light illumination (Vis) and FLD 366 nm after the SOS-Umu-C bioassay (F).


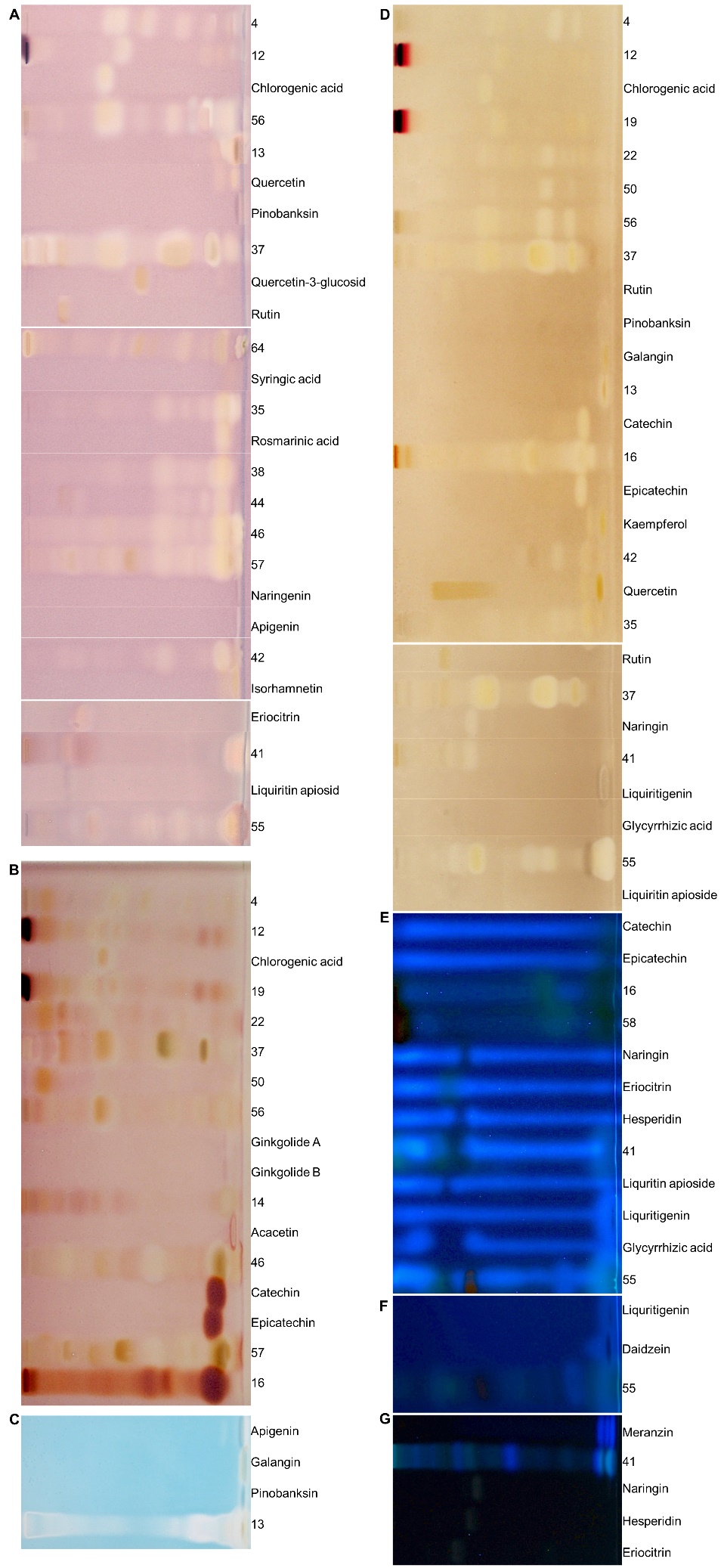


Supplementary Figure 8. NP-HPTLC−UV/Vis/FLD−EDA profiles of the plant extracts (4 µL/band, assignments in Table 1) co-chromatographed with standards (5 µL/band, 1 µg/µL) on HPTLC plate silica gel 60 F_254_ MS-grade with ethyl acetate – toluene – formic acid – water (16:4:3:2, *V/V/V/V*), up to 70 mm, detected at white light illumination (A–D) and FLD 366 nm (E–F) and after the BChE (A), β-glucosidase (B), β-glucuronidase (C) and tyrosinase (D) inhibition assays as well as pYAES (E) and pYES (F) bioassays.

Supplementary Table 1. NP-HPTLC-EDA-RP-HPLC-DAD-HESI-MS confirmation with standards and respective activity (X) in the AChE/BChE (C), α-/β-glucosidase (D), β-glucuronidase (E), and tyrosinase (F) inhibition assays as well as pYES (H) and pYAES (J) bioassays.

| **Substance** | ***hR*_F_ (±1)** | **RT [min]** | **UV λ_max_ [nm]** | ***m/z*** | **Mass signal** | **Found in** | **C** | **D** | **E** | **F** | **H** | **J** |
| --- | --- | --- | --- | --- | --- | --- | --- | --- | --- | --- | --- | --- |
| Acacetin | 98 | 8.31 | 268, 330 | 283  285 | [M−H]^−^  [M+H]^+^ | Rosemary (no. 46) |  | X |  |  |  |  |
| Apigenin | 98 | 7.79 | 239, 267, 339 | 269  271 | [M−H]^−^  [M+H]^+^ | Oregano (no. 42) | X |  | X |  |  |  |
| Catechin | 94 | 5.16 | 279 | 289  325  403  291  329 | [M−H]^−^  [M+Cl]^−^  [M+TFA−H]^−^  [M+H]^+^  [M+K]^+^ | Guarana (no. 16) |  | X |  | X |  | **X** |
| Chlorogenic acid | 37 | 3.59 | 217, 324 | 353  355  372  377  393 | [M−H]^−^  [M+H]^+^  [M+NH_4_]^+^  [M+Na]^+^  [M+K]^+^ | Artichoke (no. 4)  Fruit tea, red (no. 12)  Hibiscus (no. 19)  Elder blossom (no. 22)  Siberian ginseng (no. 56) | X | X |  | X |  |  |
| Daidzein | 98 | 7.32 | 248 | 253  255  277  293 | [M−H]^−^  [M+H]^+^  [M+Na]^+^  [M+K]^+^ | Licorice (no. 55) |  |  |  |  | X |  |
| Epicatechin | 93 | 6.02 | 203, 279 | 289  325  403  291  313  329 | [M−H]^−^  [M+Cl]^−^  [M+TFA−H]^−^  [M+H]^+^  [M+Na]^+^  [M+K]^+^ | Guarana (no. 16) |  | X |  | X |  | **X** |
| Eriocitrin | 26 | 6.59 | 284 | 595  631  658  709  597  614  619  635 | [M−H]^−^  [M+Cl]^−^  [M+NO_3_]^−^  [M+TFA−H]^−^  [M+H]^+^  [M+NH_4_]^+^  [M+Na]^+^  [M+K]^+^ | Orange peel (no. 41) | X |  |  |  |  |  |
| Galangin | 99 | 8.35 | 209, 266 | 269  271 | [M−H]^−^  [M+H]^+^ | Galangal (no. 13) |  |  | X | X |  |  |
| Ginkgolide A | 95 | 6.95 | 224  272 | 407  443  467  815  426  431  447 | [M−H]^−^  [M+Cl]^−^  [M+H_3_C-COO]^−^  [2M−H]^−^  [M+NH_4_]^+^  [M+Na]^+^  [M+K]^+^ | Ginkgo (no. 14) |  | X |  |  |  |  |
| Ginkgolide B | 95 | 6.95 | 224, 272 | 423  459  537  442  447  463 | [M−H]^−^  [M+Cl]^−^  [M+TFA−H]^−^  [M+NH_4_]^+^  [M+Na]^+^  [M+K]^+^ | Ginkgo (no. 14) |  | X |  |  |  |  |
| Glycyrrhizic acid | 25 | 7.84 | - | 410  821  861 | [M−2H]^−^  [M−H]^−^  [M+K]^+^ | Licorice (no. 55) |  |  |  | **X** |  | X |
| Hesperidin | 31 | 6.92 | 285 | 609  645  611  623  649 | [M−H]^−^  [M+Cl]^−^  [M+H]^+^  [M+Na]^+^  [M+K]^+^ | Orange peel (no. 41) |  |  |  |  |  | X |
| Isorhamnetin | 97 | 7.79 | - | 315  317 | [M−H]^−^  [M+H]^+^ | Oregano (no. 42) | X |  |  |  |  |  |
| Kaempferol | 95 | n.d. | n.d. | n.d. | n.d. | Supposed in Oregano (no. 42) |  |  |  | X |  |  |
| Liquiritigenin | 98 | 7.21 | 219, 231, 276 | 255  257  279 | [M−H]^−^  [M+H]^+^  [M+Na]^+^ | Licorice (no. 55) |  |  |  | X | X |  |
| Liquiritin apiosid | 23 | 6.65 | 217,  276 | 549  585  612  663  551  568  573  589 | [M−H]^−^  [M+Cl]^−^  [M+NO_3_]^−^  [M+TFA−H]^−^  [M+H]^+^  [M+NH_4_]^+^  [M+Na]^+^  [M+K]^+^ | Licorice (no. 55) | X |  |  | **X** |  | X |
| Naringenin | 97 | 7.51 | 226,  290 | 271  273 | [M−H]^−^  [M+H]^+^ | Oregano (no. 42) | X |  |  |  |  |  |
| Naringin | 33 | 6.87 | 284 | 579  615  639  642  581  603  619 | [M−H]^−^  [M+Cl]^−^  [M+H_3_C-COO]^−^  [M+NO_3_]^−^  [M+H]^+^  [M+Na]^+^  [M+K]^+^ | Orange peel (no. 41) |  |  |  | X |  | X |
| Pinobanksin | 99 | 7.53 | 214,  291 | 271 | [M−H]^−^ | Galangal (no. 13) | X |  | X | X |  |  |
| Quercetin | 97 | 7.40 | 255, 371 | 301  603  303 | [M−H]^−^  [2M−H]^−^  [M+H]^+^ | Supposed in Galangal (no. 13) 🡪 rebutted | X |  |  | X |  |  |
| Quercetin-3-glucosid | 54 | 6.86 | 256,  356 | 463  465  487  503 | [M−H]^−^  [M+H]^+^  [M+Na]^+^  [M+K]^+^ | Supposed in Yerba mate, green (no. 37) 🡪 rebutted | X |  |  |  |  |  |
| Rosmarinic acid | 90 | 6.18 | 218, 326 | 359  395  719  361  378  383  399 | [M−H]^−^  [M+Cl]^−^  [2M−H]^−^  [M+H]^+^  [M+NH_4_]^+^  [M+Na]^+^  [M+K]^+^ | Marjoram (no. 35)  Lemon balm (no. 38)  Peppermint (no. 44)  Rosemary (no. 46)  Thyme (no. 57) | X |  |  |  |  |  |
| Rutin | 19 | 6.84 | 256, 356 | 609  611  633  649 | [M−H]^−^  [M+H]^+^  [M+Na]^+^  [M+K]^+^ | Yerba mate, green (no. 37) | X |  |  | X |  |  |
| Syringic acid | 94 | 4.80 | 213,  264 | 197 | [M−H]^−^ | Supposed in Hawthorn (nos. 62-64) 🡪 rebutted | **X** |  |  |  |  |  |
